# Clinical features, functional consequences, and rescue pharmacology of missense *GRID1* and *GRID2* human variants

**DOI:** 10.1093/hmg/ddad188

**Published:** 2023-11-07

**Authors:** James P Allen, Kathryn B Garber, Riley Perszyk, Cara T Khayat, Steven A Kell, Maki Kaneko, Catherine Quindipan, Sulagna Saitta, Roger L Ladda, Stacy Hewson, Michal Inbar-Feigenberg, Chitra Prasad, Asuri N Prasad, Leah Olewiler, Weiyi Mu, Liana S Rosenthal, Marcello Scala, Pasquale Striano, Federico Zara, Tyler W McCullock, Robin-Tobias Jauss, Johannes R Lemke, David M MacLean, Cheng Zhu, Hongjie Yuan, Scott J Myers, Stephen F Traynelis

**Affiliations:** Department of Pharmacology and Chemical Biology, Emory University School of Medicine, 1510 Clifton Rd., Atlanta, GA 30322, United States; Center for Functional Evaluation of Rare Variants (CFERV), Emory University School of Medicine, 1510 Clifton Rd., Atlanta, GA 30322, United States; Department of Human Genetics, Emory University School of Medicine, 615 Michael St., Atlanta GA 30322, United States; EGL Genetics, 2460 Mountain Industrial Blvd., Tucker, GA 30084, United States; Department of Pharmacology and Chemical Biology, Emory University School of Medicine, 1510 Clifton Rd., Atlanta, GA 30322, United States; Department of Biomedical Engineering, Georgia Institute of Technology, 313 Ferst Drive, Atlanta, GA 30332, United States; Department of Pharmacology and Chemical Biology, Emory University School of Medicine, 1510 Clifton Rd., Atlanta, GA 30322, United States; Department of Chemistry, Emory University School of Medicine, 1515 Dickey Dr, Atlanta, GA 30322, United States; Division of Genomic Medicine, Department of Pathology, Children’s Hospital Los Angeles, 4650 Sunset Blvd, Los Angeles, CA 90027, United States; Center for Personalized Medicine, Children’s Hospital Los Angeles, 4650 Sunset Blvd, Los Angeles, CA 90027, United States; Center for Personalized Medicine, Children’s Hospital Los Angeles, 4650 Sunset Blvd, Los Angeles, CA 90027, United States; Division of Clinical Genetics, Departments of Human Genetics, OBGYN and Pediatrics, David Geffen School of Medicine at UCLA, 200 Medical Plaza, Los Angeles, CA 90095, United States; Division of Human Genetics, Department of Pediatrics, Penn State College of Medicine, 600 University Dr, Hershey, PA 17033, United States; Department of Genetic Counselling, The Hospital for Sick Children and Department of Molecular Genetics, University of Toronto, 1 King's College Circle, Toronto, ON M5G 1X8, Canada; Division of Clinical & Metabolic Genetics, The Hospital for Sick Children and Pediatrics, University of Toronto, 555 University Avenue, Toronto ON M5G 1X8, Canada; Department of Pediatrics (Section of Genetics and Metabolism), Western University and Schulich School of Medicine and Dentistry, Children’s Hospital LHSC, 800 Commissioners Road East, London, ON N6A5W9, Canada; Division of Pediatric Neurology, Department of Pediatrics and Clinical Neurological Sciences, Western University and Schulich School of Medicine and Dentistry, Children’s Hospital LHSC, 800 Commissioners Road East, London, ON N6A5W9, Canada; Department of Pediatrics, Division of Medical Genetics, University of Mississippi Medical Center, 2500 N. State St., Jackson, MS 39216, United States; Department of Genetic Medicine, Johns Hopkins University, 600 N. Wolfe St., Baltimore MD 21287, United States; Department of Neurology, Johns Hopkins University, 601 N. Caroline St., Baltimore MD 21287, United States; Department of Neurosciences, Rehabilitation, Ophthalmology, Genetics, Maternal and Child Health, Università Degli Studi di Genova, Largo Paolo Daneo, 3, 16132 Genova GE, Italy; Pediatric Neurology and Muscular Diseases Unit, IRCCS Istituto Giannina Gaslini, Pavilion 16, Via Gerolamo Gaslini, 516147 Genoa GE, Italy; Department of Neurosciences, Rehabilitation, Ophthalmology, Genetics, Maternal and Child Health, Università Degli Studi di Genova, Largo Paolo Daneo, 3, 16132 Genova GE, Italy; Pediatric Neurology and Muscular Diseases Unit, IRCCS Istituto Giannina Gaslini, Pavilion 16, Via Gerolamo Gaslini, 516147 Genoa GE, Italy; Medical Genetics Unit, IRCCS Istituto Giannina Gaslini, Pavilion 20, Via Gerolamo Gaslini, 516147 Genoa GE, Italy; Department Pharmacology and Physiology, University of Rochester Medical Center, 601 Elmwood Avenue, Rochester NY, 14642, United States; Institute of Human Genetics, University of Leipzig Medical Center, Philipp-Rosenthal-Str. 55, Haus W, Leipzig 04103, Germany; Institute of Human Genetics, University of Leipzig Medical Center, Philipp-Rosenthal-Str. 55, Haus W, Leipzig 04103, Germany; Department Pharmacology and Physiology, University of Rochester Medical Center, 601 Elmwood Avenue, Rochester NY, 14642, United States; Department of Biomedical Engineering, Georgia Institute of Technology, 313 Ferst Drive, Atlanta, GA 30332, United States; Department of Pharmacology and Chemical Biology, Emory University School of Medicine, 1510 Clifton Rd., Atlanta, GA 30322, United States; Center for Functional Evaluation of Rare Variants (CFERV), Emory University School of Medicine, 1510 Clifton Rd., Atlanta, GA 30322, United States; Department of Pharmacology and Chemical Biology, Emory University School of Medicine, 1510 Clifton Rd., Atlanta, GA 30322, United States; Center for Functional Evaluation of Rare Variants (CFERV), Emory University School of Medicine, 1510 Clifton Rd., Atlanta, GA 30322, United States; Department of Pharmacology and Chemical Biology, Emory University School of Medicine, 1510 Clifton Rd., Atlanta, GA 30322, United States; Center for Functional Evaluation of Rare Variants (CFERV), Emory University School of Medicine, 1510 Clifton Rd., Atlanta, GA 30322, United States; Emory Neurodegenerative Disease Center, 615 Michael St., Emory University School of Medicine, Atlanta, GA 30322, United States

**Keywords:** GluD1, GluD2, *GRID1*, *GRID2*, delta receptors, cerebellar ataxia, cerebellar atrophy, Cbln2, Cbln4, lurcher, schizophrenia

## Abstract

*GRID1* and *GRID2* encode the enigmatic GluD1 and GluD2 proteins, which form tetrameric receptors that play important roles in synapse organization and development of the central nervous system. Variation in these genes has been implicated in neurodevelopmental phenotypes. We evaluated *GRID1* and *GRID2* human variants from the literature, ClinVar, and clinical laboratories and found that many of these variants reside in intolerant domains, including the amino terminal domain of both *GRID1* and *GRID2*. Other conserved regions, such as the M3 transmembrane domain, show different intolerance between *GRID1* and *GRID2*. We introduced these variants into GluD1 and GluD2 cDNA and performed electrophysiological and biochemical assays to investigate the mechanisms of dysfunction of *GRID1/2* variants. One variant in the *GRID1* distal amino terminal domain resides at a position predicted to interact with Cbln2/Cbln4, and the variant disrupts complex formation between GluD1 and Cbln2, which could perturb its role in synapse organization. We also discovered that, like the *lurcher* mutation (GluD2-A654T), other rare variants in the *GRID2* M3 domain create constitutively active receptors that share similar pathogenic phenotypes. We also found that the SCHEMA schizophrenia M3 variant GluD1-A650T produced constitutively active receptors. We tested a variety of compounds for their ability to inhibit constitutive currents of GluD receptor variants and found that pentamidine potently inhibited GluD2-T649A constitutive channels (IC_50_ 50 nM). These results identify regions of intolerance to variation in the *GRID* genes, illustrate the functional consequences of *GRID1* and *GRID2* variants, and suggest how these receptors function normally and in disease.

## Graphical abstract

**Figure:**
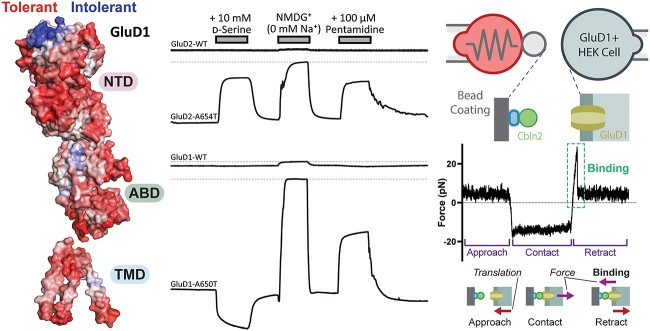

## Introduction

The GluD or delta subunits belong to a subfamily of glutamate receptors that includes the GluD1 and GluD2 receptors, which are encoded by the *GRID1* and *GRID2* genes, respectively. Both GluD1 and GluD2 are widely expressed throughout the brain, with GluD2 most strongly expressed in the cerebellum [1, 2]. GluD2 is expressed in the Purkinje cell spines and is often co-expressed with other glutamatergic receptors [1, 3, 4]. GluD1 and GluD2 proteins assemble as homomeric tetramers and contain an agonist binding domain that can bind D-serine [5, 6]. Unlike other ionotropic glutamate receptors, the GluD receptor is not discernibly gated by agonist binding alone, and may require the accessory proteins Cbln and Neurexin to form functional d-serine or glycine-responsive receptors [7]. Cbln and Neurexins, together with GluD proteins, form a trans-synaptic complex [8–11], and this complex linking the presynaptic terminal (Cbln and Neurexin) and postsynaptic density can control the recruitment of postsynaptic AMPA and NMDA receptors through the GluD1 C-terminal motifs [12]. GluD2 and Cbln1 are critical for proper cerebellar granule and Purkinje cell dendritic morphology and synapse stability [13–15].

Although genetic studies have linked *GRID1* copy number variants (CNVs) with autism [16–18] and single nucleotide polymorphisms (SNPs) in the gene have been implicated in both schizophrenia and bipolar disorder [19–22], a clear relationship between missense *GRID1* variants and clinical phenotypes is not yet well established. Transgenic animal models provide insight into potential roles of GluD1 in neurological disease. Deletion of GluD1 in mice leads to hyperactivity, social deficits, aggression and depression-like behavior, as well as deficits in learning and memory [6, 23, 24]. Adult mice lacking GluD1 in medial prefrontal cortex (mPFC) and hippocampus show a higher dendritic spine count [25], which may have implications for disorders such as autism for which a higher number of spines has been observed [26].

Loss-of-function variation in *GRID2* has been implicated in an autosomal recessive syndrome with cerebellar ataxia, eye movement abnormalities, cerebellar atrophy, and global developmental delay [27–32]. In addition, a deletion affecting the *GRID2* gene has been associated with schizophrenia [33]. Transgenic mice with *Grid2* knockout (the *hotfoot* strain) exhibit ataxia, impaired locomotion, and Purkinje cell abnormalities, and implicate GluD2 receptor involvement in cerebellar synaptic long term depression [13, 34], as well as cerebellar synaptic organization [35]. Evidence in mice and humans also suggests that missense variation in *GRID2* is involved in neurological disease pathogenesis. The *lurcher* mouse contains a *Grid2* variant [36, 37], p.Ala654Thr, which results in constitutively open ion channels that pass inward currents at rest [37], and lead to subsequent apoptosis of cerebellar Purkinje neurons [38]. Electrophysiological studies of this variant determined that the constitutively active GluD2-A654T receptor can be inhibited by d-serine [5], and potentiated by extracellular Ca^2+^ [39, 40]. In addition, p.Ala654Thr and p.Ala654Asp heterozygous *GRID2* variants were identified in patients from different families with congenital spinocerebellar ataxia, and either heterozygous or homozygous missense p.Leu656Val variants in *GRID2* have been reported in multiple patients from a large family with cerebellar ataxia [41]. These variants were speculated to result in a gain-of-function leading to a dominant or semi-dominant inheritance pattern [41]. All three variants reside in the third membrane domain (M3) motif of GluD2 (residues SYTANLA**A**F**L**), which is highly conserved and critical for glutamate receptor channel gating [42, 43]. Two of these variants alter the same amino acid as in the *lurcher* mouse (Ala654) and this strain exhibits degeneration of cerebellar Purkinje cells and ataxic gait [37]. In the *GRIA3* gene encoding another glutamate receptor (GluA3), a variant at the homologous amino acid as *lurcher* was identified in two patients with developmental delay and a disturbed sleep–wake cycle (*GRIA3* p.Ala653Thr; SYTANLA**A**FL). This change stabilizes the closed state of the AMPA receptor [44]. A recurrent *GRIA1* variant at the same location (p.Ala636Thr; SYTANLA**A**FL) in five patients is associated with intellectual disability and autism [45]. Variants at this position in *GRIN1* have also been described (p.Ala653Gly, ClinVar).

Variants in genes that encode proteins (Cbln, neurexin families) that form a trans-synaptic complex with GluD receptors may contribute to neuropsychiatric conditions through alteration in the actions of GluD1 and GluD2 receptors. Association studies showed an enrichment of heterozygous *NRXN1-NRXN3* variants in cases *versus* controls and thus linked common variation in these genes to schizophrenia, autism spectrum disorder, and Tourette’s syndrome [46]. Rare variation in *NRXN1* causes a recessive, severe epileptic encephalopathy [47]. *CBLN2* is implicated in Tourette’s syndrome [48]. Furthermore, functional transgenic animal studies suggest that *CBLN2* plays a role in compulsive behaviors and spine formation in the prefrontal cortex [49, 50]. These actions likely involve alterations in GluD receptor signaling.

Given the prominent roles in CNS development, overall intolerance to variation of the *GRID* genes (Supplementary Table S1) and accumulating evidence of their role in neurological disorders, we sought to establish critical domains and residues in GluD1 and GluD2 in order to facilitate clinical variant classification in these genes. We also anticipated analysis of variant effects could provide clues to the enigmatic function of the GluD receptor family. Here we report a comprehensive set of potential disease-associated *GRID2* and *GRID1* human variants, which includes several newly identified variants as well as a compilation of the previously published and publicly available variants. We used multiple computational and functional assays to establish the domains that are intolerant to variation and the residues that are critical for GluD1-Cbln2 protein interactions. We also assessed the function of several of the GluD1 and GluD2 variants in electrophysiological and biochemical assays and performed pharmacological studies to identify small molecules capable of modulating constitutive currents produced by *GRID* variants as potential therapeutic strategies.

## Results

### Clinical features of *GRID1* and *GRID2* human variants

*GRID1* and *GRID2* show substantial genetic intolerance to variation, as measured by residual variance intolerance scores (RVIS; Supplementary Table S1). To understand the variation that may disrupt gene function, we identified known missense, nonsense, and deletion variants in *GRID1* and *GRID2* from the public database ClinVar, the *Schizophrenia* Exome Sequencing Meta-analysis (SCHEMA) database, and published reports in the literature. In addition, a network of clinical colleagues identified new patient-derived *GRID1* and *GRID2* variants via whole exome sequencing, and we worked with individual clinical testing labs to identify *GRID1* and *GRID2* variants in patients who received exome sequencing. Clinical and genetic information describing the 57 *GRID1* (31 this study, 1 literature, 1 SCHEMA, 24 ClinVar) and 70 *GRID2* (3 this study, 5 published, 62 ClinVar) missense variants identified in patients or present in clinical databases for this study are presented in Table 1 and Supplementary Tables S2–S5.

**Table 1:** The GluD1 M3 domain is more tolerant to variation than GluD2.

Gene	Nucleotide Change	Protein Change	Domain	PolyPhen	gnomAD Alleles	gnomAD noneuro Alleles	SCHEMA Case Alleles	SCHEMA Control Alleles	Source
***GRID1***	c.1911G > T	p.Trp637Cys	M3	probably damaging	2/248 908	2/206 130	1/48 496	2/194 644	ClinVar
***GRID1***	c.1940C > T	p.Ser647Phe	M3	probably damaging	3/249 524	2/206 728	0	1/194 644	gnomAD v2.1.1
***GRID1***	c.1946C > T	p.Thr649Ile	M3	probably damaging	1/249 506	0	0	1/194 644	gnomAD v2.1.1
***GRID1***	c.1948G > A	p.Ala650Thr	M3	probably damaging	1/249 224	0	1/48 496	0	SCHEMA/gnomAD v2.1.1
***GRID1***	c.1952A > G	p.Asn651Ser	M3	benign	6/249 322	6/206 636	0	3/194 644	gnomAD v2.1.1
***GRID2***	c.1936 T > C	p.Ser646Pro	M3	probably damaging	0	0	0	0	ClinVar
***GRID2***	c.1945A > G	p.Thr649Ala	M3	probably damaging	0	0	0	0	This study
***GRID2***	c.1945A > T	p.Thr649Ser	M3	probably damaging	0	0	0	0	ClinVar
***GRID2***	c.1946C > T	p.Thr649Met	M3	probably damaging	22/281 822	19/228 708	0	0	gnomAD v2.1.1
***GRID2***	c.1948G > C	p.Ala650Pro	M3	probably damaging	0	0	0	0	ClinVar
***GRID2***	c.1949C > T	p.Ala650Val	M3	probably damaging	0	0	0	0	ClinVar
***GRID2***	c.1961C > G	p.Ala654Gly	M3	probably damaging	0	0	0	0	ClinVar
***GRID2***	c.1960G > A	p.Ala654Thr	M3	probably damaging	0	0	0	0	Coutelier *et al.* [41]
***GRID2***	c.1961C > A	p.Ala654Asp	M3	probably damaging	0	0	0	0	Coutelier *et al*. [41]
***GRID2***	c.1966C > G	p.Leu656Val	M3-S2	probably damaging	0	0	0	0	Coutelier *et al*. [41]

Nucleotide and protein changes are represented as the consequence on the canonical NCBI RefSeq for *GRID1* (NM_017551.2) and *GRID2* (NM_001510.4). A complete list of *GRID1* and *GRID2* variants with clinical information is given in Supplementary Tables S2 and S3. Variants present in ClinVar are summarized in Supplementary Tables S4 and S5. All variants were predicted by SIFT to be deleterious (not shown).

There was no common phenotype evident for individuals with *GRID1* missense variants. That is, we found little overlap in phenotypic characteristics between patients with *GRID1* variants (Supplementary Tables S2–S5). Unlike *GRID1* where phenotypes were variable, *GRID2* missense and nonsense variants presented with ataxia (n = 20) and cerebellar atrophy (n = 6), consistent with the phenotypic characteristics of mutant *Grid2 lurcher* mice [37]. Other common phenotypes were eye movement disorders (n = 10) and to a lesser degree developmental delay (n = 7). Whereas both the GluD1 and GluD2 proteins are expressed in a variety of brain regions including the cerebral cortex, hippocampus, and cerebellum, GluD2 expression is highest in the cerebellum and present at higher levels than GluD1 [1], potentially explaining the occurrence of ataxia and cerebellar atrophy in patients with *GRID2* variants.

### Regional intolerance of the *GRID1* and *GRID2* amino terminal and transmembrane domains

**Figure 1:**
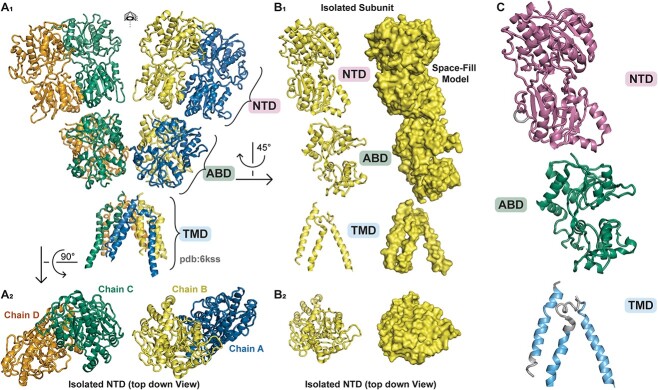
GluD1 protein structure described by Burada *et al*. (2020), pdb ID 6KSS (*see*  Supplementary Data 1). (A_1_) Full view of protein structure with four separate GluD1 protein subunits visible (chain A, *blue*; chain B, *yellow*; chain C, *green*; chain D, *orange*). (A_2_) Top down view of the amino-terminal domain (NTD) of the GluD1 tetramer. (B_1-2_) Isolated GluD1 subunit shown with ribbon and space fill model. (C) GluD1 protein NTD in *pink*, agonist-binding domain [56] in *green*, and transmembrane domain (TMD) in *cyan*.

**Figure 2:**
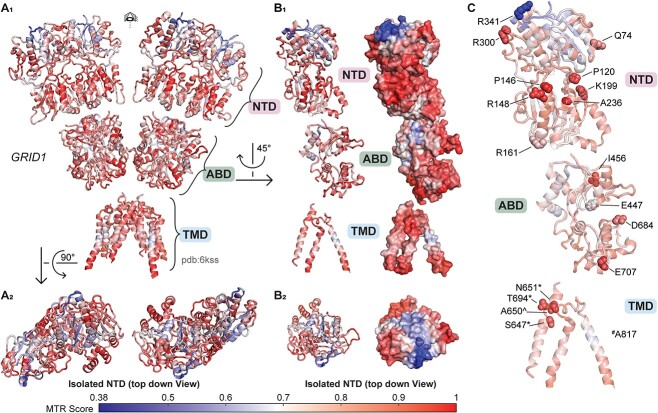
Genetic regional intolerance to variation of *GRID1.* The 3D MTR analysis for *GRID1* was developed from the GluD1 structure described by Burada *et al*. (2020) with 3D MTR residue window of 31 (see Supplementary Data 1 and 2). Low 3D MTR scores (*blue*) indicate residues less tolerant to variation, while high scores indicate residues that are more tolerant to variation [81]. (A_1_) Full view of *GRID1* 3D MTR scores on GluD1 protein structure. (A_2_) Top down view of the amino-terminal domain (NTD) of *GRID1* 3D MTR scores on GluD1 protein structure. (B_1-2_) Isolated subunit *GRID1* 3D MTR scores shown with ribbon and space fill model. (C) *GRID1* variants clinically relevant or tested here that are not present in gnomAD 2.1.1 from Supplementary Tables S2 and S4 shown in context of 3D MTR scores. ^#^ denotes position of missense variants residing at listed positions that are not represented in the displayed figure due to lack of electron density. ^*^ denotes that variants were evaluated in this study. ^^^ denotes that variants at listed positions are found in gnomAD (v2.1.1) evaluated because they were in SCHEMA patients.

**Figure 3:**
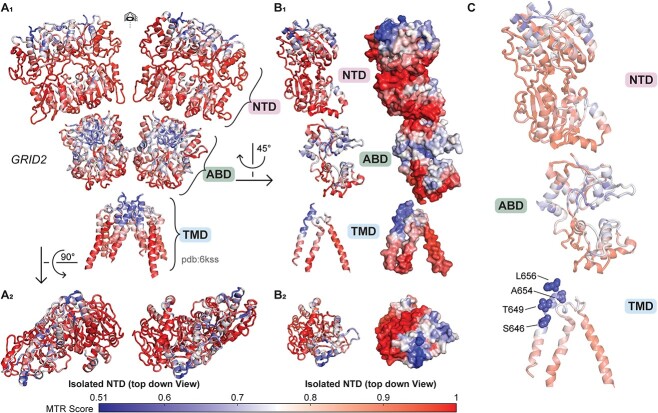
Genetic regional intolerance to variation of *GRID2*. The 3D MTR analysis for *GRID2* was developed from the GluD1 structure described by Burada *et al*. (2020) with 3D MTR residue window of 31 (see Supplementary Data 1 and 2). Low 3D MTR scores (*blue*) indicate residues less tolerant to variation, while high scores [81] indicate residues that are more tolerant to variation. (A_1_) Full view of *GRID2* 3D MTR scores mapped onto the GluD1 protein structure, assuming that GluD2 has a similar architecture to GluD1. (A_2_) Top down view of the amino-terminal domain (NTD) of *GRID2* 3D MTR scores on the GluD1 protein structure. (B_1-2_) Isolated subunit *GRID2* 3D MTR scores shown with ribbon and space fill model. (C) *GRID2* variants clinically relevant or tested here that are not present in gnomAD v2.1.1 from Supplementary Tables S3 and S5 shown in context of *GRID2* 3D MTR scores.

The missense tolerance ratio (MTR) is a parameter that estimates the subregions within gene coding domains that are under purifying selection based on population genetics data [51]. The MTR quantifies the frequency of missense variants present in a sliding window of the protein coding domain observed in the human population reported by the Exome Aggregation Consortium Database (ExAC). Since the GluD1 and GluD2 receptors have complex tertiary and quaternary structure (Fig. 1), we used a 3D MTR algorithm that can predict hotspots of regional intolerance by evaluating nearest neighbors in 3-dimensional space within the protein structure rather than nearest neighbors along the polypeptide linear chain [52]. Using this approach, we determined that both GluD1 and GluD2 receptors have regions of the amino terminal domain (NTD) that are intolerant to genetic variation, such as the most extracellular portion of the NTD (Figs 2–3; see also Supplementary Fig. S4). Interestingly, 7 *GRID1* missense variants reported here (GluD1-P120Q, GluD1-R161C, GluD1-K199R, GluD1-A236S, GluD1-R300L, GluD1-R341Q, GluD1-P146L, R148C; Fig. 2) and two variants in ClinVar (GluD1-Q117E, GluD1-P146L) that are absent from gnomAD reside within the intolerant NTD domain (GluD1 residues 21-421; Supplementary Tables S2 and S3), suggesting potentially clinically-relevant NTD variants may exist for GluD1 receptors. Four missense variants reported in ClinVar that reside in the GluD2 NTD (GluD2 residues 24–425) were absent from gnomAD (Supplementary Table S5). Additional patient-derived variants in the NTD are also present in gnomAD, although most are outside of the most intolerant regions (Supplementary Fig. S4).

While there was strong overlap in intolerant regions in the NTD across both genes, we also identified several differences in regional intolerance between the *GRID1* and *GRID2*. For example, GluD2 showed regional intolerance around the extracellular end of the M3 transmembrane helix close to the linker regions, whereas GluD1 showed no such intolerance. Overall 9/70 of the clinically reported *GRID2* missense variants were absent from the gnomAD database (Table 1, Fig. 3) and resided in the M3 transmembrane helix. By contrast only 1/56 of the *GRID1* patient variants resides in M3, and there are multiple variants in this region found in gnomAD v2.1.1 (Table 1, Supplementary Tables S2 and S4, Fig. 3). This suggests differential roles of the M3 helix or different functional responses to genetic variation between the GluD1 and GluD2 receptors. The GluD1 M4 domain also appears intolerant to variation using the 3D MTR method, and one variant (GluD1-A817T) from a patient with developmental delay and other neurological features was identified in this region and was absent from the gnomAD database. Both GluD1 and GluD2 agonist binding domains revealed some regional intolerance, which could suggest important roles for Ca^2+^ binding and d-serine binding in these receptors, as missense mutations in this domain can alter these properties [40]. Interestingly, it appears that the agonist binding domain for GluD2 is less tolerant to variation than that of GluD1, raising the possibility that ligand binding to GluD2 may be more important for its biological role(s) than perhaps ligand binding to GluD1 is for its role(s).

### NTD variants predicted to disrupt interactions with Cbln1 and Cbln2

**Figure 4:**
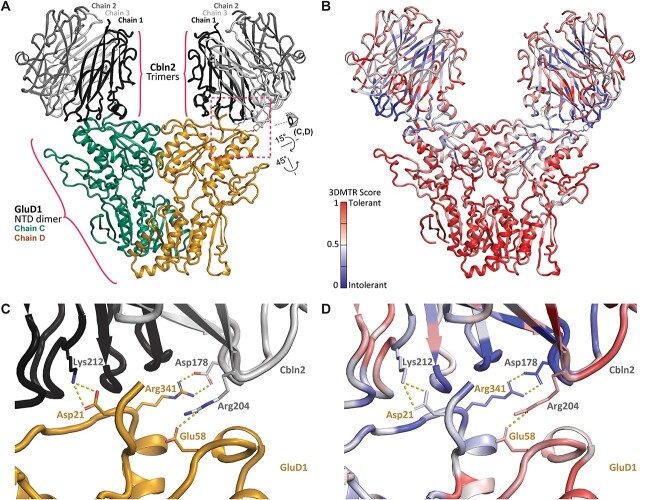
GluD1-Cbln protein-protein interactions. (A) Side view of a homology model (*see*  Supplementary Data 1) of human *GRID1* in complex with human *CBLN2*. This structure was one frame from the MD simulation modeling the interactions between these proteins (Supplementary Fig. S2). (B) 3D MTR analysis of *CBLN2* and *GRID1* using the closest 21 residues in the analysis (*see*  Supplementary Data 2). Low MTR scores (*blue*) indicate residues intolerant to variation, while high scores indicate residues tolerant to variation [81]. (C) Interacting residues between the GluD1-Cbln2 complex. Labeled residues are predicted to form both hydrogen bonding and salt bridge interactions between GluD1 (Asp21, Arg341, Glu58) and Cbln2 (Lys212, Asp178, Arg204). R341-D178, E58-R204, and D21-K212 salt bridges are predicted to occur during 99.5%, 99.5%, and 100% of analyzed frames. View shown is depicted in panel A by the eye cartoon. On the chain not shown here, R341 interacts with a D176. (D) 3D MTR of the site of GluD1-Cbln2 interaction (same view as in C).

The NTD of the GluD1 and GluD2 receptors binds the presynaptically-secreted cerebellins to form trans-synaptic complexes with pre-synaptic neurexins [9, 11, 53]. Presynaptic binding of the neurexin-cerebellin complex to GluD1 transduces signals that control the recruitment of AMPA and NMDA receptors to the postsynaptic membrane [12]. Thus, the intolerance observed in the GluD1 and GluD2 NTD may be due to its binding to the Cbln1-neurexin trans-synaptic protein complex [8–11]. That is, variants in the *GRID1* and *GRID2* genes could be disease-associated if they alter the binding affinity to Cbln1 or Cbln2. Because 8 of the reported missense *GRID1* variants reside in the NTD, we generated a homology model of GluD1-Cbln2 interactions to look at possible mechanisms for variant dysfunction. The model predicts that three GluD1 amino side chains (Asp21, Glu58, Arg341) form salt bridges between GluD1 and Cbln2. Cbln2 Ser345 participates in sidechain H-bond interactions, and Trp343 and Met346 form main chain H-bond interactions (Fig. 4). Importantly, Arg341 is also the site of the human variant *GRID1* p.R341Q (Fig. 2C). If these residues participate in protein complex formation *in vivo*, then changing the positively charged arginine to the neutral glutamine at position 341 should diminish the strength of this interaction and alter the binding affinity between GluD1 and Cbln2. We ran 3D MTR analysis on the GluD1-Cbln2 homology model to look at the intolerance of *GRID1* and *CBLN2* near the site of the protein–protein interactions, and found a marked intolerance of both genes at sites encoding residues near their respective sites of interaction (Fig. 4B), including the GluD1-R341Q variant (Fig. 2C). While the full scope of how GluD1 works *in vivo* remains incomplete, this model and intolerance analysis emphasize the importance of both the GluD1-Cbln2 protein–protein interaction and genetic variants in these domains.

While little is known about the phenotypic features of *GRID1* human variants, GluD1 knock-out mice exhibit social deficits, in addition to anxiety-like, depression-like and aggressive behaviors [6, 23, 24]. These behaviors may be consistent with the phenotypic features of this patient with GluD1-R341Q, which include intellectual disability, ADHD, aggression, anxiety, and schizoaffective disorder. These clinical correlates together with our prediction that this variant perturbs interactions between GluD1 and cerebellins within an intolerant region suggests this variant may have deleterious effects on the trans-synaptic GluD1 function.

### Human variant R341Q reduces the interaction between the GluD1-NTD and Cbln2

**Figure 5:**
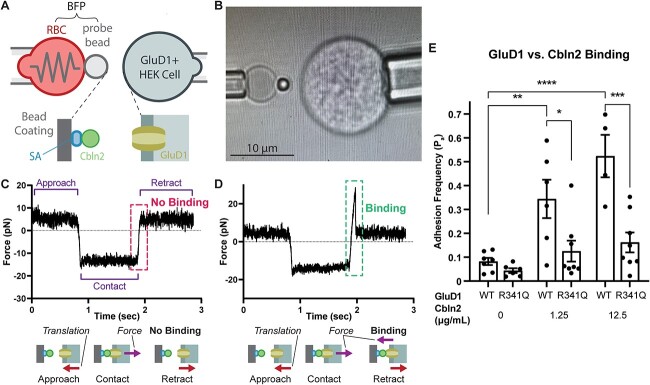
GluD1-R341Q reduces binding between GluD1-NTD and Cbln2. (A) Schematic of the biomembrane force probe (BFP) experiment. (B) Photomicrograph of the BFP experiment. (C) Force trace of a single BFP trial where there was no GluD1/Cbln2 interaction, as demonstrated by the lack of a tension force (+) upon probe retraction. (D) Force trace of a single BFP trial where there was a GluD1/Cbln2 interaction as evidence by the positive force reading upon probe retraction. (E) Adhesion frequencies between HEK293T cells expressing similar levels of GluD1 WT or R341Q (Supplementary Fig. S5) and BFP beads coated with indicated concentrations of Cbln2. Each point represents an adhesion frequency evaluated from the observed number of binding events divided by the total number of repeated contacts (50-100) between a single pair of cell and bead. Also shown are mean ± SEM for each condition. Abbreviations: RBC, red blood cell; SA, streptavidin. Means were compared with an unpaired ANOVA with post hoc Tukey test, where ^*^^*^^*^^*^ indicates *P* < 0.0001, ^*^^*^^*^P < 0.001, ^*^^*^P < 0.01, and ^*^P < 0.05. Only pertinent significant comparisons are shown.

Given that the human variant GluD1-R341Q is in a portion of the receptor that is highly intolerant and predicted to interact with Cbln2, it should have a strong functional effect. However, this residue is distal to the agonist binding domain and the channel pore, and thus seems unlikely to alter their actions. We hypothesized that given its position and likely interaction with Cbln2, GluD1-R341Q should disrupt this protein–protein interaction. To study the interaction between GluD1 and Cbln2, we performed a biomembrane force probe (BFP) experiment where Cbln2 was attached to a probe bead that is manipulated to touch a HEK293T cell expressing GluD1 (Fig. 5A and B). In this experiment the probe bead is attached to a red blood cell, which acts as a spring to gauge the forces during measurements, and held using a micropipette by suction. The HEK cell was repeatedly brought into contact with the probe bead to enable interaction between GluD1 and Cbln2, as indicated by a compressive force (Fig. 5C and D, negative force), and then it was pulled away to observe whether interaction indeed occurred. In some cases the force simply returned from negative to zero, and this indicates that the contact did not produce binding between GluD1 and Cbln2 (Fig. 5C). In other cases a tension force was observed (positive force), indicating adhesion (Fig. 5D). Such contact-retraction cycle was repeated 50-100 times per bead-cell pair to enumerate a frequency of the occurrence of the positive events and multiple bead-cell pairs were tested to tabulate the individual adhesion frequency for each condition. To avoid complications from endogenous GluD1 expression, GluD1 KO HEK293 cells were used (Supplementary Fig. S3). As expected, increasing concentrations of Cbln2 resulted in an increase in the adhesion frequency for GluD1-WT expressing cells (Fig. 5E). By contrast, the adhesion frequency was reduced with R341Q in all scenarios where Cbln2 was applied to the probe bead suggesting that the propensity for binding was disrupted by the GluD1 variant. Control experiments confirmed that WT and variant receptors were expressed at similar levels (Supplementary Fig. S5). These data strongly suggest that this NTD variant can alter the interaction of GluD1 and Cbln2, and raises the possibility that the intolerance of this region to variation reflects disruption of the interactions between GluD receptors and cerebellins.

### Constitutive activity of *GRID1* and *GRID2* human transmembrane domain variants

**Figure 6:**
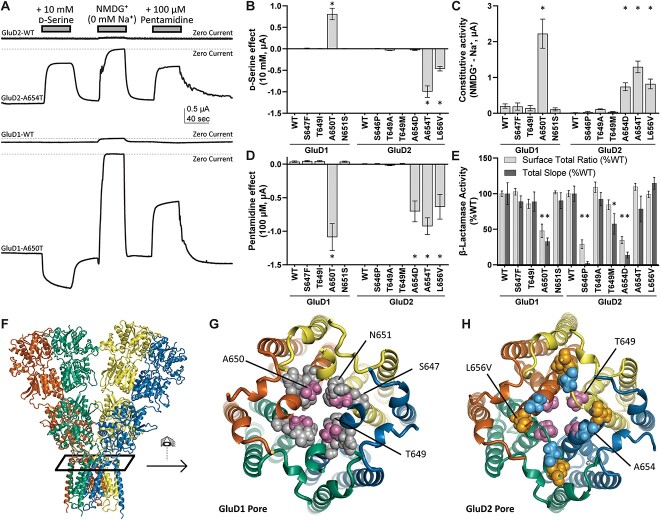
Screen for constitutive channel activity of *GRID1* and *GRID2* human variants. (A) Two electrode voltage clamp assay in *Xenopus laevis* oocytes expressing cRNA encoding *GRID1* and *GRID2* human variants. The zero current line is shown (*dashed line*). (B) Changes in current upon application of 10 mM d-serine. Positive values represent potentiation of constitutive current, while negative values are inhibition of constitutive current. (C) Constitutive current determined by ionic substitution of Na^+^ for NMDG^+^. (D) Application of 100 μM pentamidine inhibits constitutively active GluD1 and GluD2 variant receptors. (E) β-Lactamase-GluD1 and -GluD2 protein fusion constructs were assayed to determine surface expression of GluD1 and GluD2 variant receptors (see Methods). Values are normalized to wild-type surface/total ratios, and wild-type total protein expression. (F) GluD protein structure (GluD2 homology model) indicating the view in (G) and (H). (G) GluD1 ion channel pore (homology model) and (H) GluD2 ion channel pore (homology model) showing the localization of constitutively active variants in the channel pore. ^*^P < 0.05 ANOVA with Dunnett’s multiple comparisons test.

The M3 transmembrane helix plays an essential role in gating for most members of the glutamate receptor family of AMPA, kainate, and NMDA receptors. Moreover, the GluD2 *lurcher* mutation within the highly conserved SYTANLAAF motif of the M3 transmembrane helix produces constitutively active channels [37]. We therefore tested whether patient-derived variants in the *GRID2* M3 domain (GluD2-L656V, GluD2-T649A, GluD2-A654T, GluD2-A654D, GluD2-S646P) as well as gnomAD variants in M3 (GluD2-T649M) could produce constitutive currents (see Table 1). We introduced these 6 variants into rat GluD2 cDNA and performed voltage clamp recordings from *Xenopus* oocytes injected with variant cRNA. In order to determine whether the expression of these constructs produced constitutive inward currents through the GluD receptors, we designed an assay that evaluated the dependence of constitutive current flow on the presence of extracellular permeant ions, a conventional means by which to assess ion channel activity. The external solutions either contained permeant Na^+^ or equimolar concentrations of the impermeant cation NMDG^+^ (see Methods). This ionic replacement experiment allowed the quantification of differences in the constitutive influx of sodium ions through presumably open GluD2 variant receptors, which we refer to as a difference current. We found that there was a minimal difference current between NMDG^+^- and Na^+^-containing solution for oocytes expressing wild type GluD2 receptors (18 ± 1.6 nA, n = 14, Fig. 6, Table 2). Oocytes expressing the previously characterized GluD2 *lurcher* mutation (p.A654T) had a large difference current between NMDG^+^- and Na^+^-containing solution of 1300 ± 160 nA (n = 14), confirming the constitutive activation of these GluD2 ion channels. Two previously reported human variants GluD2-A654D and GluD2-L656V also produced large constitutive currents of 740 ± 110 nA (n = 12) and 1040 ± 140 nA (n = 12), respectively. While not significant by Dunnett’s multiple comparisons test, the GluD2-T649A variant, which is newly reported in this study, showed a difference current of 113 ± 14 nA (n = 13; see Table 2). By contrast, GluD2-T649M, which was present in gnomAD, showed minimal constitutive currents (37 ± 11 nA, n = 16).

**Table 2:** Screen for constitutive activity of *GRID1* and *GRID2* human variants.

	Constitutive activity (NMDG^+−^ Baseline) nA (n)	d-Serine effect^a^ (Baseline −10 mM d-Serine) nA (n)	Pentamidine effect^a^ (Baseline −100 μM Pentamidine) nA (n)
**GluD1-WT**	200 ± 60 (13)	2.3 ± 2.9 (13)	37 ± 11 (14)
**GluD1-R341Q**	230 ± 130 (6)	7.2 ± 1.8 (6)	64 ± 15 (6)
**GluD1-S647F**	190 ± 100 (10)	14 ± 3.8 (10)	−47 ± 11 (10)
**GluD1-T649I**	140 ± 76 (9)	8.9 ± 1.2 (9)	−45 ± 9.5 (9)
**GluD1-A650T**	2220 ± 410 (12)^*^	810 ± 130 (12)^*^	−1090 ± 200 (12)^*^
**GluD1-N651S**	100 ± 43 (10)	7.6 ± 1.6 (10)	38 ± 8.3 (10)
**GluD2-WT**	18 ± 1.6 (14)	−3.6 ± 1.0 (14)	3.6 ± 4.8 (14)
**GluD2-D535E**	56 ± 23 (12)	−5.8 ± 2.4 (12)	−0.67 ± 1.1 (12)
**GluD2-S646P**	28 ± 23 (11)	5.2 ± 6.0 (11)	6.5 ± 5.4 (11)
**GluD2-T649A**	113 ± 14 (13)	−35 ± 5.6 (14)	−18 ± 4.8 (14)
**GluD2-T649M** ^b^	37 ± 11 (16)	−0.31 ± 2.8 (16)	5.8 ± 7.0 (16)
**GluD2-A654D**	740 ± 110 (12)^*^	−29 ± 10 (14)	−700 ± 160 (14)
**GluD2-A654T** ^c^	1300 ± 160 (14)^*^	−1000 ± 130 (14)^*^	−930 ± 130 (14)^*^
**GluD2-L656V**	1040 ± 140 (12)^*^	−460 ± 53 (12)^*^	−640 ± 190 (12)^*^
**GluD2-R710W**	27.7 ± 2.3 (11)	−2.3 ± 0.60 (11)	0.82 ± 1.9 (11)

Data are mean ± SEM (n).

^*^Indicates *P* < 0.05, One way ANOVA, Dunnett’s multiple comparisons test.

^a^Positive values indicate an increase of activity and negative values indicate a decrease of activity.

^b^This was found in gnomAD.

^c^This is the constitutively active *lurcher* mutation.

Because 3DMTR predicted the M3 transmembrane helix within GluD1 to be tolerant to variation, and multiple M3 *GRID1* variants are reported in gnomAD, variation in GluD1 M3 might not produce constitutive activity, in contrast to what we observed for GluD2. We performed a parallel assay for constitutive activity of the single *GRID1* M3 variant found in the SCHEMA schizophrenia database, as well as three additional *GRID1* M3 variants in presumably healthy individuals from the gnomAD database. None of the three *GRID1* variants from gnomAD produced constitutive activity, consistent with the 3D MTR prediction that this conserved region of *GRID1* is tolerant to change. However, the *GRID1* SCHEMA variant GluD1-A650T produced strong constitutive activity (2220 ± 410 nA, n = 12, *P* < 0.0001 Fig. 6, Table 2). This variant, which appears in an individual with schizophrenia in the SCHEMA database (1/48496 alleles), is absent from both SCHEMA’s control population (0/194644 alleles) and the gnomADv2.1.1 non-neuro population database, a more stringent subset curated to remove variants from patients with neurological disease (see Table 1). This suggests a more localized intolerance to variation in this domain of GluD1 M3 compared to GluD2 and demonstrates a new functional mechanism by which *GRID1* variants might trigger neurological disease. As a control to ensure constitutive activity is due to the location of the M3 residues, GluD1-R341Q, which resides in an intolerant region but in the NTD rather than M3, was also tested for constitutive activity. As expected, given that this variant is distal to the pore and presumably participates in transynaptic binding of cerebellins, we did not detect any constitutive activity from GluD1-R341Q currents (Table 2).

### Surface expression of *GRID1* and *GRID2* human variants

The results from these TEVC experiments suggest substantial differences in the degree of constitutive activity between variants in both *GRID1* and *GRID2* (Fig. 6, Table 2). To assess whether the variable degree of constitutive activity observed for *GRID1* and *GRID2* M3 variants reflected altered surface trafficking, we used a colorimetric β-lactamase reporter assay in GluD-transfected HEK293 cells to quantify the surface to total protein expression ratios. We found that constitutive activity for GluD1-A650T correlated with decreases in both the surface/total ratio and total protein expression (Table 3; Fig. 6E). Similar decreases were not observed for GluD1 variants without constitutive activity, including GluD1-R341Q (Table 3). By contrast, there was less consistency in results for the *GRID2* variants. Whereas GluD2-A654D had reduced total protein expression and GluD2-T649M exhibited a slight reduction in total and surface protein expression (Table 3), other variants with constitutive activity did not show reduced surface/total or total protein expression (GluD2-T649A, GluD2-A654T, and GluD2-L656V).

**Table 3:** Surface expression of *GRID1* and *GRID2* variants determined by β-lactamase activity.

	Constitutive activity	Surface/Total, %WT (n)	Total, %WT (n)
**GluD1-WT**	—	100 ± 4.7 (8)	100 ± 15 (8)
**GluD1-R341Q**	—	108 ± 6.6 (5)	75 ± 16 (5)
**GluD1-S647F**	—	103 ± 5.6 (9)	89 ± 8.0 (9)
**GluD1-T649I**	—	86 ± 7.9 (9)	89 ± 14 (9)
**GluD1-A650T**	**present**	48 ± 12 (5)^*^	33 ± 11 (5)^*^
**GluD1-N651S**	—	102 ± 2.6 (6)	90 ± 11 (6)
**GluD2-WT**	—	100 ± 6.4 (21)	100 ± 11 (21)
**GluD2-D535E**	—	95 ± 9.9 (8)	63 ± 16 (8)^*^
**GluD2-S646P**	—	29 ± 9.3 (3)^*^	1.9 ± 2.6 (3)^*^
**GluD2-T649A**	**likely**	109 ± 11 (9)	92 ± 9.3 (9)
**GluD2-T649M**	—	84 ± 10 (9)	57 ± 14 (9)^*^
**GluD2-A654D**	**present**	35 ± 7.5 (8)^*^	14 ± 4.2 (8)^*^
**GluD2-A654T**	**present**	109 ± 7.5 (11)	78 ± 18 (11)
**GluD2-L656V**	**present**	99 ± 6.9 (7)	115 ± 8.4 (7)
**GluD2-R710W**	—	111 ± 7.2 (10)	93 ± 7.0 (10)

Data are mean ± SEM (n). See Fig. 6 for related graph.

^*^ *P* < 0.05 one way ANOVA, with Dunnett’s multiple comparisons test, all variants compared to WT (normalized to 100%).

We hypothesize that reduced expression of GluD1-A650T and GluD2-A654D might be a consequence of the large constitutive leak currents produced by these channels, which can trigger Ca^2+^-mediated toxicity in mammalian cells showing strong protein expression [54]. However, GluD2-A654T also showed large leak currents and exhibited no deficits in surface trafficking, which may point to changes in calcium permeability that could influence cell toxicity. Of all *GRID1* and *GRID2* variants that showed no constitutive current, only the *GRID2* variant GluD2-S646P showed reduced surface and total protein expression, which might account for the receptor’s lack of constitutive current despite the variant location in the M3 pore-forming transmembrane helix. The proline in this transmembrane region may produce a completely non-functional or misfolded protein, causing pathogenicity via a separate mechanism.

### Constitutively active *GRID* human variants are modulated by D-serine and Ca^2+^

**Figure 7:**
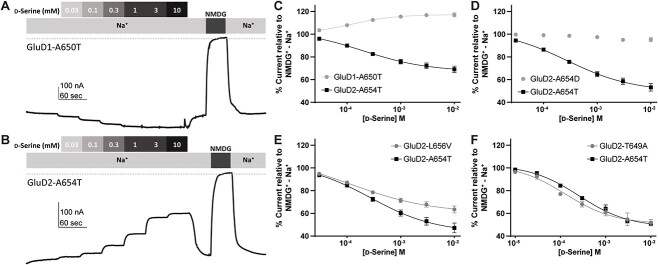
Modulation of constitutively active GluD1 and GluD2 M3 variants by d-serine. Representative concentration-response curves showing d-serine modulation of the constitutive current observed for (A) GluD1-A650T and (B) GluD2-A654T. The differences between the baseline current and the current in NMDG^+^ are used to establish theoretical maximum inhibition for each recording. The dotted line represents zero current. (C–F) D-Serine concentration-response curve for d-serine modulation of constitutively active M3 variants GluD1-A650T (C), GluD2-A654D (D), GluD2-L656V (E) and GluD2-T649A. For each experiment, same day control curves recorded for the *lurcher* variant GluD2-A654T are shown. Unlike GluD2-A654T, GluD2-L656V, GluD2-T649A, we observed that GluD1-A650T is modestly potentiated by d-serine rather than inhibited. A654D shows a near complete loss of d-serine potency.

Previous studies had shown that when d-serine occupies the agonist binding domain of GluD2, it induces closure of the bilobed domain around d-serine, similar to binding of agonists to other glutamate receptors [5]. Furthermore, d-serine reduces the constitutive current in GluD2-A654T, suggesting that d-serine-mediated agonist binding domain closure could inhibit gating produced by constitutively active M3 variants. We therefore applied 10 mM d-serine in Na^+^ external solution (before replacement with NMDG^+^) to determine whether it triggered agonist domain closure, as measured via changes in constitutive receptor activity. Whereas d-serine had minimal activity on the GluD2-WT difference current, it reduced by ~50% the maximum possible activity of two variants that were constitutively active (GluD2-A654T and GluD2-L656V), as demonstrated by the Na^+^ -NMDG^+^ difference current (Fig. 6). These findings were consistent with previous studies on the effect of d-serine on the GluD2-A654T *lurcher* mutation [5], and suggest that GluD2-L656V shares a similar gain-of-function mechanism. Figure 7 shows the concentration-dependence of d-serine inhibition of GluD2-A654T and GluD2-L656V, which has a similar trend to d-serine inhibition of a variant that produces constitutive baseline current (GluD2-T649A). All three of these variants GluD2-A654T, GluD2-L656V, and GluD2-T649A showed similar potencies for d-serine of 100–300 μM IC_50_ (Fig. 7E and F, Table 4). By contrast, d-serine had little effect on constitutively active GluD2-A654D (Fig. 7D, Table 4).

**Table 4:** Modulation of constitutive current from *GRID1* and *GRID2* variants expressed in *Xenopus* oocytes by d-Serine and **c**alcium.

	d-serine EC_50_ μM [95% CI] (n)	d-serine % Max (NMDG^+−^ Na^+^) Mean ± SEM (n)	d-serine EC_50_ A654T/EC_50_ variant	Ca^2+^ EC_50_ μM [95% CI] (n)	Ca^2+^ % Max (NMDG^+−^ Na^+^) Mean ± SEM (n)	Ca^2+^ EC_50_ A654T/EC_50_ variant
**GluD1-A650T**	130^a^ [90, 160] (11)	120 ± 1.8 (11)^*^	—	290^b^ [140, 360] (10)	65 ± 3 (10)^*^	—
**GluD2-A654T**	360 [300, 390] (32)	51 ± 1.3 (32)	1.0	150 [140, 160] (32)	299 ± 5.8 (32)	1.0
**GluD2-T649A**	210 [120, 300] (7)	49 ± 2.5 (7)	1.7	290 [250, 310] (10)	345 ± 55 (10)	0.52
**GluD2-L656V**	240 [170, 280] (14)	62 ± 3.2 (14)^*^	1.5	170 [150, 190] (11)	260 ± 9.9 (11)^*^	0.88
**GluD2-A654D**	> 10 000 (11)	85 ± 2.6 (15)^*^	—	170^b^ [92, 230] (12)	82 ± 10 (12)^*^	0.88
**GluD2-A654T-R710W** ^c^	270 [220, 300] (14)	42 ± 3.5 (14)	1.3	80 [76, 85] (9)	245 ± 15 (9)	1.88
**GluD2-A654T-D535E** ^c^	700 [550, 790] (15)	60 ± 2.2 (15)	0.51	5600^b^ [3080, 6500] (15)	70 ± 2.0 (15)^*^	—

EC_50_ data are mean [95% CI Lower, Upper] (number of oocytes), where the confidence intervals were determined from log EC_50_ values. The maximal effect of d-serine is given as a mean ± SEM percent of the constitutive leak current, determined as the difference current between NMDG^+^ and Na^+^ containing solutions. See Figs 4 and 5 for related concentration-response curves.

^*^ *P* < 0.05 two-sample Welches t-test with Bonferroni correction comparison with GluD2-A654T control recorded under the same conditions.

^a^Potentiated by d-serine rather (not inhibited); see d-serine % Max (NMDG^+−^ Na^+^).

^b^Inhibited by Ca^2+^ (not potentiated); see Ca^2+^ % Max (NMDG^+^ − Na^+^) data column.

^c^Double mutation including A654T to generate constitutively active channels in a variant that previously demonstrated no constitutive activity.

**Figure 8:**
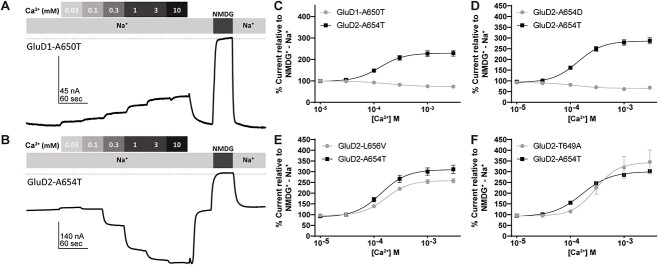
Modulation of constitutively active GluD1 and GluD2 M3 variants by extracellular Ca^2+^. Representative Ca^2+^ concentration-response curves for (A) GluD1-A650T and (B) GluD2-A654T. The dotted line represents zero current. (C–F) Ca^2+^ concentration-response of GluD1-A650T, GluD2-A654D, GluD2-L656V, GluD2-T649A, and GluD2-A654T. For each experiment, same day control curves recorded for the *lurcher* variant GluD2-A654T are shown. All recordings normalized to NMDG^+^ − Na^+^ difference current. Unlike GluD2-A654T, GluD1-A650T and GluD2-A654D were modestly inhibited by Ca^2+^.

As shown by Naur and colleagues [5], extracellular calcium potentiates the GluD2 constitutive activity [39, 40]. To further explore the properties of the newly identified constitutively active GluD2-A654D, GluD2-L656V, and GluD2-T649A, we assessed the concentration-dependence of Ca^2+^ potentiation on these receptors (Fig. 8). Two of these variants (GluD2-L656V, GluD2-T649A) had similar potencies for Ca^2+^ potentiation within a range of 0.1–0.4 mM EC_50_ (Fig. 8E and F, Table 4). In addition, Ca^2+^ acted as a weak inhibitor of GluD2-A654D (Fig. 8D, Table 4). These findings suggest that constitutively active GluD2 variant receptors respond to the endogenous modulators d-serine and Ca^2+^, but not always in the same manner.

Like GluD2 receptors, GluD1 receptors are modulated by d-serine and Ca^2+^ [55]. Therefore, we assessed the constitutively active currents generated by GluD1-A650T for d-serine and Ca^2+^ sensitivity. Surprisingly, unlike GluD2-A654T, GluD1-A650T is slightly potentiated by d-serine and inhibited by Ca^2+^ (Fig. 7C, Fig. 8C). Despite having opposite effects, the EC_50_ values for d-serine and Ca^2+^ acting on GluD1-A650T were 120 μM and 290 μM, respectively, which is within the same range for GluD2-A654T (d-serine EC_50_ 360 μM, Ca^2+^ EC_50_ 150 μM; Table 4). As a control, we assessed GluD1-R341Q, which—as expected due to its location in the distal NTD—was insensitive to D-serine (Table 2).

### Human *GRID2*variants in the agonist binding domain alter d-serine and Ca^2+^ potency

**Figure 9:**
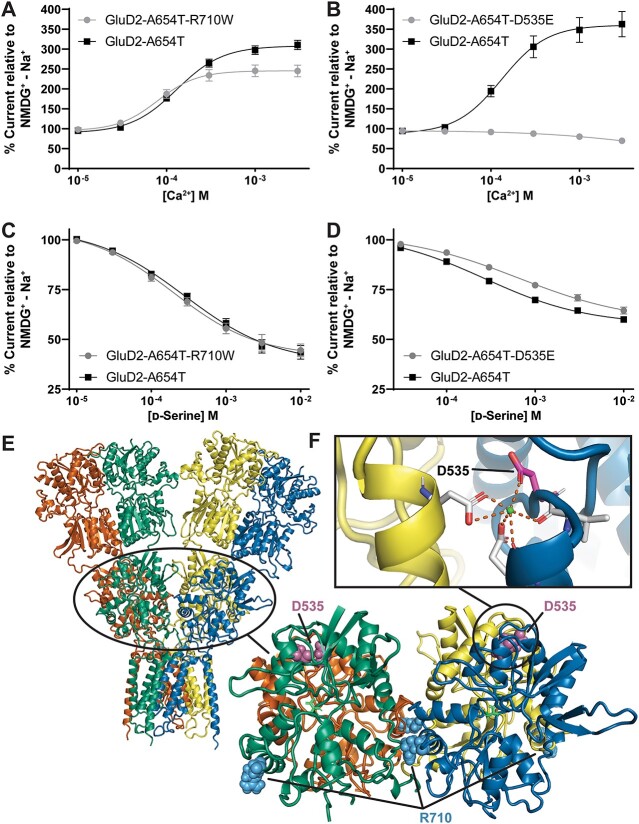
Non-constitutively active *GRID2* agonist binding domain variants alter d-serine and Ca^2+^ potency. For each experiment, same day control curves recorded for the *lurcher* variant GluD2-A654T are shown. (A) Ca^2+^ concentration-response curve for inhibition of GluD2-A654T-R710W and -A654T (B) Ca^2+^ concentration-response curve for inhibition of GluD2-A654T, D535E and GluD2-A654T. Unlike GluD2-A654T, GluD2-A654T,D535E is weakly inhibited by Ca^2+^. (C) d-Serine concentration-response of GluD2-A654T, R710W and GluD2-A654T. (D) d-Serine concentration-response of GluD2-A654T,D535E and GluD2-A654T. (E) GluD2 protein structure (GluD2 homology model) with a closer view of the GluD2 agonist binding domain shows the location of GluD2-R710W and GluD2-D535E. (F) Variant GluD2-D535E is at a site that is important for Ca^2+^ binding (PBD: 2V3T).

While many *GRID2* variants demonstrated constitutive activity, this was not the case for all variants tested. Although GluD2-R710W in the agonist binding domain segregated with disease in three siblings with cerebellar ataxia and delayed psychomotor development in a homozygous state [56] (Supplementary Table S3), it did not show constitutive currents when expressed in *Xenopus* oocytes (Table 3) or alter surface expression (Table 3). We also identified another agonist binding domain missense variant present in gnomAD (*GRID2* c.1605C > G, p.Asp535Glu; gnomAD: heterozygous 1/250696 Alleles) that alters one of the residues involved in the coordination of Ca^2+^ binding to the GluD2 receptor (Fig. 9) [40]. We confirmed that GluD2-D535E did not produce constitutive currents (Table 2) and did not alter receptor surface expression (Table 3), as expected given its location in the agonist binding domain.

To allow us to investigate d-serine and Ca^2+^ interactions with the agonist binding domain, we introduced the constitutive GluD2-A654T variant into GluD2-R710W and GluD2-D535E to convert these variants to constitutively active receptors. GluD2-A654T, D535E variant receptors modestly reduced d-serine potency, showing a 1.9-fold shift of the EC_50_ compared to that observed for GluD2-A654T alone (Fig. 9). Additionally, GluD2-A654T, R710W showed increased Ca^2+^ potency compared to GluD2-A654T controls (i.e. decreased EC_50_, Fig. 9, Table 4). By contrast, GluD2-A654T, D535E receptor showed a complete loss of Ca^2+^ potentiation and was instead slightly inhibited by Ca^2+^ (Fig. 9). These changes in function could indicate a potential mechanism of pathogenicity of these variants, although the role of Ca^2+^ regulation of native GluD2 receptors is still poorly understood *in vivo*. Additionally, the GluD2-R710W variant stabilizes the closed conformation of the GluD2 agonist binding domain [57], providing an additional possible clue for disease pathogenesis, although the mechanism of dysfunction of GluD2 agonist binding domain variants remains to be determined.

### Inhibition of GluD1/2 constitutive current by FDA-approved channel blockers

d-Serine is one of the first compounds shown to inhibit the constitutively active current of the GluD2-A654T (GluD2 *lurcher)* mutation through its association with the agonist binding domain of the receptor [5]. However, d-serine is not sufficiently potent (IC_50_ of about 0.3 mM) to consider therapeutic treatment. Other studies have evaluated similar compounds at the GluD2 *lurcher* mutation, but none were more active than d-serine [58]. We explored whether the constitutively active current produced by GluD2-T649A might also be inhibited by some of these compounds, and thus evaluated other amino acids for their potency to inhibit GluD2-T649A and GluD2-A654T activity (Supplementary Fig. S6, Supplementary Table S8). Glycine, l-aspartic acid, and d-alanine inhibited both GluD2-T649A and GluD2-A654T (Supplementary Table S8). The largest potency difference was in l-aspartic acid, with a 19-fold increase in potency for GluD2-T649A (0.27 mM) compared to GluD2-A654T (5.0 mM) (Supplementary Table S6). However, like the GluD2-A654T variant, none of these compounds were more effective on GluD2-T649A than d-serine, which showed modest dependence on Ca^2+^ (Supplementary Figs S6 and S7).

**Figure 10:**
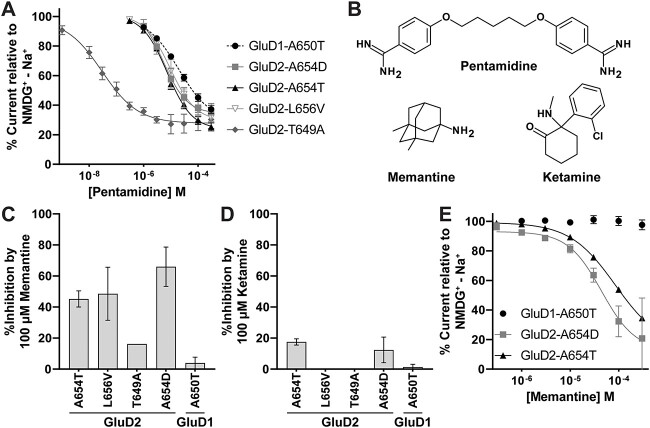
Rescue pharmacology of GluD1 and GluD2 constitutively active variants using FDA-approved channel blockers in *Xenopus* oocytes. (A) Pentamidine composite concentration-response curves for GluD2 variants show that pentamidine has an IC_50_ value that is > 200 fold lower (i.e. more potent) for GluD2-T649A (IC_50_ 37 nM) than for GluD2-A654T. Recordings were performed in 1 mM Ca^2+^. (B) Structures of compounds tested in this study are shown. (C and D) Results are shown for a single concentration screen for memantine (C) and ketamine (D) inhibition of GluD receptor channel constitutive activity. (E) Memantine concentration-response curves are shown for GluD2-A654D and GluD2-A654T.

In addition to ligands that bind within the agonist binding domain, we also tested the glutamate receptor channel blocker pentamidine [59] for its ability to inhibit the constitutive currents generated by GluD2 M3 variants. Pentamidine has also been shown to inhibit the GluD2-A654T receptor with an IC_50_ of 11 μM [60]. We tested pentamidine on GluD2-A654T and GluD2-T649A and found that pentamidine had a ~200-fold increase in potency on GluD2-T649A (IC_50_ 36 nM) compared to GluD2-A654T (IC_50_ 9.6 μM; Fig. 10A, Table 5). Compared to GluD2-A654T, pentamidine was more potent (3.7 μM), GluD2-L656V showed no significant difference in pentamidine potency (10 μM), and GluD1-A650T showed reduced pentamidine potency (20 μM; Fig. 10A, Table 5). We conclude that pentamidine inhibition is a feature of all four of these variants, but with differing potencies spanning two orders of magnitude. These diverse pharmacological properties provide an opportunity to evaluate potential precision medicine approaches to identify compounds used for the treatment of specific GluD2 receptor variants.

**Table 5:** Channel blockers of constitutively active GluD1 and GluD2 current.

	Pentamidine IC_50_ (μM)	Memantine IC_50_ (μM)
**GluD1-A650T**	20 [16, 23] (10)	> 300
**GluD2-A654T**	9.6 [7.1, 11] (13)	89 [60, 104] (14)
**GluD2-T649A**	0.036 [0.014, 0.039] (15)	ND
**GluD2-L656V**	10 [6.7, 12] (12)	ND
**GluD2-A654D**	3.7 [2.6, 4.4] (13)	55 [31, 87] (5)

Data are mean [95% CI Lower, Upper] (n), where confidence intervals were determined from log IC_50_ values. *See*  Fig. 10 for related concentration inhibition curves. ND indicated no data is available.

We additionally assessed the effect of the FDA-approved NMDAR channel blockers memantine and ketamine on constitutively active GluD1 and GluD2 receptor variants (Fig. 10B). We found that memantine inhibits GluD receptor constitutively active currents more effectively than ketamine (Fig. 10C and D). GluD2-A654T and GluD2-A654D were inhibited by memantine with an IC_50_ of 89 μM and 55 μM, respectively (Fig. 10D, Table 5). Though memantine’s potency on these receptors is apparently too low to present an effective therapeutic treatment, this demonstrates that compounds in this class could also be considered as potential therapies.

## Discussion

Here we present a comprehensive description of *GRID1* and *GRID2* variants that have been reported in individuals with neurological disorders, in addition to a number of newly described variants. Clinical interpretation of variation in these genes is impeded by the low number of cases reported and the general lack of understanding of the critical residues and domains of the GluD proteins, as well as lack of mechanistic understanding of GluD signaling. In this work, we sought to advance the understanding of these proteins through systematic analyses of clinically identified variants in computational and functional assessments. While we cannot confirm the pathogenicity of most of these *GRID1* variants, predictions of regional intolerance and gene level intolerance increase the likelihood that these *GRID1* variants could be pathogenic, particularly those arising in the distal amino terminal domain. Functional evaluation shows for the first time that a *GRID1* missense variant altering a residue in the NTD (GluD1-R341) can weaken the interaction of GluD1 with Cbln2, creating a precedent that suggests that intolerance of this region may perturb transsynaptic complex formation and associated signaling. In addition, we show that a *GRID1* SCHEMA variant showed strong constitutive activity, consistent with a potential contribution to neuropathology in this patient and a role in schizophrenia.

By contrast, *GRID2* variants demonstrated more consistent phenotypic characteristics including spinocerebellar ataxia and oculomotor symptoms, suggestive of pathogenicity, but further functional studies as well as genetic trio studies are needed. Many variants resided in the transmembrane domain, which for GluD2 (but not GluD1) is largely intolerant to variation. Electrophysiological studies showed that human GluD2 variants in the M3 transmembrane helix generate constitutive activity, as well as functional changes in d-serine inhibition and calcium potentiation of this current. All *GRID2* variants tested showed similar surface expression as WT GluD2 receptors except for GluD2-A654D, which was expressed at a lower level overall and had a lower surface/total protein ratio. While it is unclear how GluD2 signals, these changes in functional receptor characteristics could impact neuronal function in multiple ways. For example, the constitutive current observed with some variants constitutes a gain-of-function that could provide a constant depolarization, and might allow sufficient Ca^2+^ entry to engage mechanisms that lead to cell death [37]. Moreover, both GluD2 expression [61, 62] and d-serine binding to GluD2 [34] are required for certain forms of cerebellar synaptic plasticity. The observation that GluD2 variants in the agonist binding domain alter d-serine and Ca^2+^ potency at constitutive receptors may suggest that these variants perturb the downstream effects of d-serine and Ca^2+^ interactions with the agonist binding domain.

We predict that the constitutive currents we observed with variants in rat GluD2 M3 mimic the effects in patients harboring those variants, as has been previously shown for the GluD2-A654T mutation in mice. Both the GluD2-A654T (*lurcher*) mutation and patient variants such as A654D, L656V, and T649A share similar constitutive activity, as observed in our voltage clamp studies, and they also share the phenotype of cerebellar ataxia. Further studies, such as those involving animal models, could elucidate the mechanism by which dysfunction of these variants produces cerebellar ataxia. We expect that for gain-of-function constitutively active GluD2 variants, a small molecule capable of inhibiting the constitutive inward current could reduce excitotoxity of the Purkinje cells [37] and possibly reduce cerebellar atrophy. This presents a potential therapy for patients with constitutively active GluD2 variants but would require considerably more cellular and molecular data to confirm, including rescue of ataxic symptoms *in vivo* using GluD2 inhibitors in constitutively active knock-in mouse models. Furthermore, any therapeutic strategies to inhibit these gain-of-function variant receptors would need to target an aberrant activity of the variant receptor (such as constitutive activation), be free of unacceptable side effects due to the disruption of the as yet unknown function of native GluD2, and have relatively few off-target liabilities.

Cryo-EM structures reported for the GluD1 [63] and GluD2 [64] receptors show a non-swapped NTD architecture that is unlike any other in the glutamate receptor family. This architecture suggests structural mechanisms by which they might signal, and thus differential roles for these receptors that could require trans-synaptic protein complex with Cbln1/Cbln2 and neurexin for signaling. Additionally, GluD1 is critical for producing electrical currents in response to noradrenaline in dorsal raphe neurons [65]. While it is not yet known how α1-adrenergic receptor activation in these neurons translates to GluD1-dependent currents, these data suggest that GluD1 might be capable of gating under physiological conditions. Despite these advances, a ligand capable of gating GluD1 or GluD2 receptors has yet to be found [66, 67]. The lack of understanding of the roles of these receptors in synaptic currents hinders functional analysis of human variants. However, understanding the localization of these missense variants in critical intolerant functional domains raises the possibility that this conservation is important for neuronal physiology, and provides important insight into how GluD receptors may participate in brain function and disease.

## Materials and methods

### Identification of variants

*GRID1* and *GRID2* variants were identified from ClinVar, the peer-reviewed literature, clinical colleagues who ordered whole exome sequencing, and from diagnostic laboratories (EGL, GeneDx). EGL provided *GRID1* and *GRID2* variants that were identified by whole exome sequencing in patients with varied symptoms. IRB approval was in place for all interactions with patients at identifying institutions.

### Synthesis of cDNA constructs

Human GluD1 (*GRID1*) and rat GluD2 (*Grid2*) cDNAs were introduced into the pGEM-HE and pCI-neo vectors. *GRID1* (NM_017551.3) gene fragments were synthesized by Integrated DNA Technologies and cloned into empty vectors using InFusion cloning (Clontech). The rat *Grid2* cDNA sequence (NM_024379.1) was cloned into the pGEM-HE vector using the same protocol; rat *Grid2* amino acid sequence is 97.52% identical to the human form (NM_001510.3), differing only in 25 positions. However, rat and human GluD2-encoding cDNA are identical in the M3 domain in which many of the variants we studied reside (see Supplementary Fig. S1). The full open reading frame for both constructs were verified by Sanger sequencing (Eurofins) and the full plasmid sequence was verified by whole plasmid sequencing (plasmidsaurus). Variant cDNA changes identified in patients were introduced into the cloned human *GRID1* (NM_017551.3) in pGEM-HE and cloned rat *Grid2* cDNA sequence (NM_024379.1) in pGEM-HE vector using Quikchange Lightning (Agilent) protocol according to the manufacturer’s instructions. Vectors were linearized and cRNA was synthesized using T7 RNA mMESSAGE kit (Invitrogen).

β-lactamase (β-lac; synthesized by Integrated DNA Technologies) fusion constructs were made by fusing the β-lactamase open reading frame into the cDNA for rat *Grid2* in pCI-Neo vector, and human *GRID1* in pCI-Neo in-frame between the signal peptide sequence and the amino terminal domain using InFusion (Clontech). Variant cDNA changes were introduced in these constructs following the same procedures described above.

### *Xenopus* oocyte two-electrode voltage clamp experiments

*Xenopus laevis* oocytes were prepared from ovaries purchased from Xenopus 1 (Dexter, MI) as previously described, [68] and incubated at 16°C in culture Barth’s solution (in mM) 88 NaCl, 2.4 NaHCO_3_, 1 KCl, 0.33 Ca(NO_3_)_2_, 0.41 CaCl_2_, 0.82 MgSO_4_, 10 HEPES, pH 7.4 with NaOH, supplemented with 1 U/ml penicillin and 1 μg/ml streptomycin. Oocytes were injected with approximately 5–40 ng RNA per oocyte and then incubated at 16–19°C for 1–3 days in culture Barth’s solution. The levels of GluD2-A654D and GluD2-A654T cRNA were reduced to 1.5 ng and 2.5 ng, respectively, due to oocyte toxicity. Two-electrode voltage clamp (TEVC) recordings were performed as previously described [69, 70]. Unless otherwise specified, extracellular solution contained (in mM) 90 NaCl, 3 KCl, 0.5 BaCl_2_, 0.01 EDTA, 10 HEPES, and was brought to pH 7.4 with NaOH. The oocytes were placed in a dual track recording chamber and gravity perfused with solution exchange controlled via a computer-driven 8-valve positioner (Digital MVP Valve). Voltage control and data acquisition were achieved with Warner OC725C amplifiers (Warner Instruments). Recording electrodes were filled with 0.3 M KCl, and currents were recorded with membrane voltage held at −40 mV. To establish constitutive activity of recombinant receptors when compared to wild type receptors, we used the oocyte recording solution described above, but with 90 mM N-methyl-D-glucamine (NMDG) chloride replacing 90 mM NaCl. The non-permeant cation NMDG^+^ does not pass through the channel, and a comparison of leak current in NMDG and Na^+^ external solution allows for determination of the amount of membrane current at rest due to sodium influx. In addition to establishing constitutive activity, 90 mM NMDG was used in recording protocols with drug treatment to obtain the percent maximum theoretical inhibition of a compound, or to determine the relative degree of drug potentiation. For Ca^2+^ concentration-response curves, choline chloride was used to maintain equimolar concentrations of Cl^−^ ions in the extracellular solution across the changing concentrations of calcium chloride. All compounds used in this study for concentration-response oocyte recordings were dissolved in deionized water as 50–100 mM stock solutions. In some rescue pharmacology experiments, we substituted 1 mM CaCl_2_ for 0.5 mM BaCl_2_ to evaluate Ca^2+^-dependent control of receptor responses.

### Determination of GluD1 and GluD2 surface expression with β-lactamase activity

HEK cells were plated in 96-well plates (50 000 cells/well) and transiently transfected (FuGENE6, Promega) 24 h later with cDNA encoding β-lac-GluD1-variant, β-lac-GluD1-WT, β-lac-GluD2-variant, or β-lac-GluD2-WT, as previously described [71]. Eight wells were transfected for each construct, and surface and total protein expression activities were each measured in quadruplicate 24 h post-transfection. For surface expression analysis, cells were washed with Hanks Balanced Salt Solution (HBSS; in mM, 140 NaCl, 5 KCl, 0.3 Na_2_HPO_4_, 0.4 KH_2_PO_4_, 6 glucose, 4 NaHCO_3_) supplemented with 10 mM HEPES (pH 7.4) and then replaced with 100 μl HBSS and 100 μM nitrocefin (Millipore) for surface activity. For total protein expression treatment groups, cells were first washed with HBSS and then were lysed in 50 μl water for 30 min, then combined with 50 μl HBSS and 200 μM nitrocefin. The plate was then warmed to 30°C and absorbance was measured every min for 30 min at 486 nm in a plate reader at 30°C. The absorbance *vs* time for each well was fit by linear regression to determine the slope, and the slope for WT vs variant β-lac expression was compared to assess surface and total protein expression.

### Data analysis

Concentration-response relationships were analyzed using Prism 8.4 (GraphPad Software) and fit by the following equations:


(1) \begin{equation*} Response\ \left(\%\right)=100/\left(1+{\left({\mathrm{EC}}_{50}/\left[ agonist\right]\right)}^{nH}\right) \end{equation*}

where EC_50_ is the agonist concentration that elicited a half maximal response, and *nH* is the Hill slope, or


(2) \begin{align*} Response\ \left(\%\right)=&\left(100- minimum\right)/\left(1+{\left(\left[ concentration\right]/{\mathrm{IC}}_{50}\right)}^{nH}\right)\nonumber \\&+ minimum \end{align*}

where *minimum* is the residual percent response in saturating concentration (constrained to be > 0) of the experimental compounds, IC_50_ is the concentration of inhibitor that causes half maximal inhibition, and *nH* is the Hill slope. Data are represented as mean ± SEM. Statistical significance of logEC_50_ or logIC_50_ was determined by one-way analysis of variance (ANOVA) with a Dunnett’s multiple comparisons post-hoc test with a significance threshold of *P* < 0.05. Sample sizes were adjusted so that the power to detect an effect size of 1 was > 0.9 (GPower 3.1). All error bars on figures represent the SEM.

### Homology Modeling

A homology model of the GluD1-Cbln2 complex was generated from 5KC9 (mouse *Grid1* NTD) with 2.3 Å resolution [8], 6KSP (rat *Grid1*, only NTD used) with 8.1 Å resolution [63], 5H4B (mouse *CBLN4*) with 2.3 Å resolution [72], and 5KCA (human *GRID2* NTD complex with human *CBLN1*) with 3.1 Å resolution [8]. The target protein sequence modelled was the NTD of the human *GRID1* (NP_060021.1) and human *CBLN2* (NP_872317.1) C1q domain (no N-terminal domain). Structural alignment of the models was performed in PyMol version 2.4 (Schrödinger, LLC) using the “align” command. First, chains A, B, C, and G from 5KCA were aligned to chain A of 6KSP, followed by chains D, E, F, and H of 5KCA to chain B of 6KSP. This achieved an alignment of the human GluD2-Cbln1 structure to the rat GluD1 structure. Next, chains A and B of 5KC9 were independently aligned to 6KSP to align mouse GluD1 NTD to the rat GluD1 structure. Next, 5H4B was aligned to chains A, B, and C of 5KCA, then a second copy of 5H4B to chains D, E, and F of 5KCA to align Cbln4 to Cbln1. Due to strong sequence homology between these structures, these structural alignments all achieved favorable root mean square deviations (RMSDs) near 2 Å. A total of 10 homology models were generated using Modeller version 10.1 [73], from which the lowest energy model based on the DOPE score was selected [74].

Protein preparation for MD simulations was performed in Schrodinger Maestro (Schrödinger Release 2021–2; Protein Preparation Wizard; Schrödinger, LLC). Hydrogen atoms were added to the model and side chain protonation states were assigned using PROPKA with the pH set to 7.4. Hydrogen bond networks were first programmatically optimized, then side chain protonation, rotamer, and tautomer states were visually inspected and manually corrected when necessary using the interactive optimizer within the Protein Preparation Wizard (Schrödinger Release 2020-4; Schrödinger, LLC). Energy minimization was performed first on hydrogen atoms only, followed by two rounds of restrained minimization of the full model. A bounding box was created around the structure with a 9 Å window between the furthest point on the model and the periodic boundary. Water molecules were added to the bounding box using SPC model and the net charge on the system was then neutralized with Na^+^, with more Na^+^ ions being added for a final concentration of 0.15 M. Restraints on the model were placed on the C-terminal end of the GluD1 proteins, the N and C-terminal ends of Cbln2. The model was run for 10 ns, at 100 ps per frame at 278°K using the NVT ensemble class. The simulation was then extended at 310°K for 400 ns. Analysis of the full simulation was run in VMD, loading in every 7 frames of the simulation. RMSD plot was generated using the RMSD trajectory VMD plugin. Subsequent analysis was conducted from frames 300 onwards with stabilized RMSD (See Supplementary Fig. S2). Hydrogen bonding percent occupancy was calculated using the HBonds VMD plugin. Salt bridges were analyzed using the Salt Bridges VMD plugin. Model pdb files are available as Supplemental Information.

Homology models without subsequent molecular dynamics of full length GluD1 and GluD2 (Fig. 6, Fig. 9) were generated using Modeller version 10.1 [74]. These models were made with GluD1 in complex with Cbln2, and GluD2 in complex with Cbln2, however this is not shown in these figures though the model is available in supplemental information as generated. Both models included templates 5KC9 (mouse *Grid1* NTD) with 2.3 Å resolution [8], 6KSS (rat *Grid1*) with 8.1 Å resolution [63], 5H4B (mouse *CBLN4*) with 2.3 Å resolution [72], and 5KCA (human *GRID2* NTD complex with human *CBLN1*) with 3.1 Å resolution [8], and 5WEO (GluA2, transmembrane domain) with 2.3 Å resolution [75]. The target sequence for GluD1-Cbln2 was human *GRID1* (NP_060021.1) (NTD-TMD) and human *CBLN2* (NP_872317.1) C1q domain (no N-terminal domain). The target sequence for GluD2-Cbln1 was human *GRID2* (NP_001501.2) (NTD-TMD) and human *CBLN1* (NP_004343.1) C1q domain (no N-terminal domain). Models are available as Supplementary Data 1.

### Three-dimensional missense tolerance ratio (3DMTR) analysis of *GRID* and *CBLN*

The calculation of the 3D MTR [52] was performed using a MATLAB (Mathworks, version R2022a) application. The executable 3D MTR application along with an operation manual explaining how to use the application on other proteins can be found at GitHub (https://github.com/riley-perszyk-PhD/3DMTR, current version v2.000). Colored pdb files with implemented 3D MTR are available as Supplemental Information. Briefly, the structure associated with GluD1 encoded by *GRID1* (PDB:6KSS, Fig. 1) was used for both *GRID1* and *GRID2* 3D MTR calculations since the *GRID2* structure had lower resolution. 3D MTR calculations for *GRID2* were performed following a sequence alignment between *GRID1* and *GRID2* within the analysis software. As there are four copies of each subunit in the structure, the scores of the four chains were averaged together to produce a single score for when the 3D MTR is displayed onto a single subunit. To aid in visualizing the span of 3D MTR score the blue-white-red color scale was determined individually for each structure. The model for a single dimer of *GRID1* NTDs and two trimers of *CBLN2* was used to calculate the intra-complex 3D MTR for the GluD1-Cbln2 interaction. 3D-MTR results are available as Supplementary Data 2.

### Generation of a GluD1 −/− HEK293T cell line

The HEK293T GluD1 −/− (GluD1 KO) cell line was generated following a similar procedure as previously described [76]. Briefly, a guide sequence (5’-CTACTCCATCAAGGTCATCG-3′) targeting the second exon of the human *GRID1* gene (NM_017551.3) was cloned into the BbsI site of the pSpCas9(BB)-2A-GFP (PX458) to generate a *GRID1*-PX458. The base PX458 vector was a generation gift form Feng Zhang (Addgene plasmid #48138) [77]. *GRID1*-PX458 was then transfected into the parental HEK293T cell line. Three days post transfection, cells were harvested with trypsin and GFP positive cells were enriched with fluorescence activated cell sorting (FACS) using a BioRad S3e Fluorescent Cell Sorter (BioRad). After sorting, GFP positive cells were diluted and plated into 96 well culture plates. One week after plating, wells that contained only a single colony of HEK cells were marked and allowed to grow to confluency within the 96 well, with fresh media being added to the well as needed. These monoclonal cell lines were than expanded and genotyping was used to confirm *GRID1* genetic disruption using the following primers: *GRID1* F1 5’-TCTGTCCTGGGATTTGGGTGGG-3′ and *GRID1* R1 5’-CCGAGAAACAAAGACTGCCCCG-3′. A single monoclonal cell line (*GRID1* KO C4, referred to here as the GluD1 KO cell line) which contained a + 1 insertion within exon 2 that ultimately results in premature stop codon within exon 3 was selected for these studies (Supplementary Fig. S3). GluD1 KO cells were subsequently used for FACs and Force probe experiments (see below).

### Re-expression of wildtype and mutant GluD1 on GluD1^−/−^ HEK293T cell line

Lentiviral plasmids were constructed by subcloning WT and variant human *GRID1* cDNA into pHRemGFP plasmids optimized for high expression [78]. Sequences were verified through Sanger sequencing (EurofinsGenomics). Lentiviruses were prepared using HEK293T cells. GluD1 KO HEK293T cells were transduced with virus expressing either WT or the human variant GluD1-R341Q and then sorted using the intracellular IRES cleaved GFP tag with flow cytometry to obtain matched expression for WT and variant receptors using immunostaining with a rabbit primary antibody against GluD1 (1:50, Glutamate Delta-1 antibody; Alomone labs) and secondary anti-rabbit AF647 antibody (ThermoFisher Scientific). Briefly, cells were detached with TrypLE (Thermofisher), neutralized with D10 media (Gibco DMEM supplemented with 1 mM sodium pyruvate, 10% FBS, 50 U/ml penicillin and 50 μg/ml streptomycin), and then washed with FACs (fluorescence-activated cell sorting) buffer (1× PBS without calcium and magnesium, 1% BSA, 25 mM HEPES, and 5 mM EDTA) before incubation with primary antibody diluted in FACs buffer for one hour, rotating at 4°C. Cells were then washed 2× with FACs buffer before incubation with secondary antibody diluted in FACs buffer for one hour at 4°C, rotating. Cells were washed two more times with FACs buffer before flow cytometry analysis.

### Generation of soluble Cerebellin 2

A lentiviral vector encoding full-length human Cerebellin 2 (Cbln2, NP_872317.1) with C-terminal 6xHIS and AVI tag for biotinylation was designed and purchased (Twist). Protein was expressed in BirA+ HEK 293 T cells previously transduced with pHR-CMV-TetO2_HA-Bir [78] for biotinylation of proteins with the addition of 100 μM D-biotin. Cbln2 protein was purified by Ni-NTA gravity column purification. Cell supernatant containing secreted protein was collected with the addition of protease inhibitor cocktail (Thermofisher; 1000× dilution) sterile-filtered through 0.45 μm filter, and then allowed to incubate 12 h rotating overnight at 4°C with Nickel Agarose (CubeBiotech). After rotating, gravity columns (Marvelgent Biosciences) were equilibrated with wash buffer (20 mM imidazole, 50 mM NaH_2_PO_4_, 300 mM NaCl in deionized H_2_0, pH 8.0) before cell supernatant was run through twice. The column was washed again with wash buffer after flow-through. and then eluted with elution buffer (500 mM imidazole, 50 mM NaH_2_PO_4_, 300 mM NaCl in deionized H_2_0, pH 8.0) that was allowed to incubate with the capped column 15 min before collection of eluted proteins. All purification steps were done at 4°C. Buffer exchange and concentration into 1× phosphate buffer saline (PBS) without calcium or magnesium was accomplished by 3× column centrifugation (10 kDa cutoff; Pall Corporation, 4°C). Protein purity was validated by SDS-page and flow cytometry (anti-HIS PE antibody 1:20, NovusBiotechnological).

### Biomembrane force probe for adhesion frequency measurements

Red blood cells (RBCs) were isolated from blood obtained by finger prick from healthy volunteers in accordance with an approved Georgia Tech IRB protocol. RBCs were biotinylated through covalent linkage to biotin-PEG3500-SGA (JenKem USA, TX), 30 min rotating at room temperature in C-buffer (80 mM Na_2_CO_3_, 126 mM NaHCO_3_). Biotinylated RBCs were then incubated with nystatin for 30 min at 4°C, washed twice, and stored in N2-5% buffer (280 mM KCl, 40 mM NaCl, 1 mM KH_2_PO_4_, 5 mM Na_2_HPO_4_, 28 mM sucrose in H_2_0, pH 7.2) for subsequent use in biomembrane force probe (BFP) experiments. Borosilicate glass beads of 2 μm in diameter (Distrilab Particle Technology) were first covalently coupled with mercapto-propyl-trimethoxy silane (United Chemical Technologies, Bristol, PA), followed by covalently linking tetravalent streptavidin (SA)-maleimide (Sigma-Aldrich, ST. Lois, MO) in Phosphate Buffer (230 mM NaH_2_PO_4_ and 200 mM Na_2_HPO_4_ in deionized water at pH 6.8) by overnight incubation at 4°C and then washed with Phosphate Buffer once more after incubation.

After incubation with sub-saturating levels (1.25 or 12.5 μg/ml) of biotinylated human Cbln2, glass beads were washed and then incubated with anti-HIS PE antibody (1:20; NovusBiotechnological) for 30 min rotating at room temperature in 1× PBS−/− plus 2% BSA. Beads were washed and then flowed in parallel with standard calibration beads (BD Quantibrite PE Beads, BD).

The BFP technique has been described previously [79]. Briefly, inside of a chamber of Leibovitz’s L-15 media (Thermofisher) with 1% BSA and 5 mM HEPES, biotinylated RBCs are aspirated with a micropipette (2 μm inner diameter). A 2 μm glass bead, conjugated with SA and coated with or without biotinylated human Cbln2 (1.25 or 12.5 μg/ml) at subsaturating level, is attached to the biotinylated RBC at the apex with a helper pipette, and is referred to as a “probe”. An opposing pipette is used to pick up the GluD1 KO HEK293T cells transduced to re-express WT or variant GluND1. The RBC with Cbln2 coated probe is aligned with the GluD1-expressing HEK293T cell, and the pipette aspirating the HEK293T cell is driven to make cyclic contacts with the probe by a piezoelectric translator (Physical Instrument, MA) with sub-nanometer precision via a capacitive sensor feedback control. A customized image analysis LabView (National Instrument, TX) program tracks the bead position with a 3-nm (standard deviation, SD) displacement precision in real-time allowing for the generation of a curve that is used to detect if either binding occurred or not [80]. Each cell pair is allowed to contact the probe for 50–100 touches and a final number of binding events out of total contacts is numerated as the adhesion frequency (*P*_a_).

## Supplementary Material

GRID_Variants_Supplemental_Revised_10-26-2023_FINAL_Rev_ddad188

Supplemental_Data_1_Models_of_GluD1_GluD2_and_Cbln2_ddad188

Supplemental_Data_2_3D_MTR_GRID1_GRID2_and_GRID1-CBLN2_interaction_NEW_ddad188

## Data Availability

The published article includes all datasets/code generated or analyzed during this study.

## References

[1] Nakamoto C, Konno K, Miyazaki T. et al. Expression mapping, quantification, and complex formation of GluD1 and GluD2 glutamate receptors in adult mouse brain. J Comp Neurol 2020;528:1003–27. 31625608 10.1002/cne.24792

[2] Lomeli H, Sprengel R, Laurie DJ. et al. The rat delta-1 and delta-2 subunits extend the excitatory amino acid receptor family. FEBS Lett 1993;315:318–22. 8422924 10.1016/0014-5793(93)81186-4

[3] Landsend AS, Amiry-Moghaddam M, Matsubara A. et al. Differential localization of delta glutamate receptors in the rat cerebellum: coexpression with AMPA receptors in parallel fiber-spine synapses and absence from climbing fiber-spine synapses. J Neurosci 1997;17:834–42. 8987804 10.1523/JNEUROSCI.17-02-00834.1997 PMC6573242

[4] Mayat E, Petralia RS, Wang YX. et al. Immunoprecipitation, immunoblotting, and immunocytochemistry studies suggest that glutamate receptor delta subunits form novel postsynaptic receptor complexes. J Neurosci 1995;15:2533–46. 7891187 10.1523/JNEUROSCI.15-03-02533.1995 PMC6578125

[5] Naur P, Hansen KB, Kristensen AS. et al. Ionotropic glutamate-like receptor delta2 binds D-serine and glycine. Proc Natl Acad Sci U S A 2007;104:14116–21. 17715062 10.1073/pnas.0703718104 PMC1955790

[6] Yadav R, Hillman BG, Gupta SC. et al. Deletion of glutamate delta-1 receptor in mouse leads to enhanced working memory and deficit in fear conditioning. PLoS One 2013;8:e60785. 23560106 10.1371/journal.pone.0060785 PMC3616134

[7] Carrillo E, Gonzalez CU, Berka V. et al. Delta glutamate receptors are functional glycine- and -serine–gated cation channels in situ. Sci Adv 2021;7:eabk2200. 34936451 10.1126/sciadv.abk2200 PMC8694607

[8] Elegheert J, Kakegawa W, Clay JE. et al. Structural basis for integration of GluD receptors within synaptic organizer complexes. Science 2016;353:295–9. 27418511 10.1126/science.aae0104 PMC5291321

[9] Tao W, Diaz-Alonso J, Sheng N. et al. Postsynaptic delta1 glutamate receptor assembles and maintains hippocampal synapses via Cbln2 and neurexin. Proc Natl Acad Sci U S A 2018;115:E5373–81. 29784783 10.1073/pnas.1802737115 PMC6003362

[10] Lee S-J, Uemura T, Yoshida T. et al. GluRδ2 assembles four neurexins into trans-synaptic triad to trigger synapse formation. J Neurosci 2012;32:4688–701. 22457515 10.1523/JNEUROSCI.5584-11.2012 PMC6622077

[11] Uemura T, Lee SJ, Yasumura M. et al. Trans-synaptic interaction of GluRdelta2 and neurexin through Cbln1 mediates synapse formation in the cerebellum. Cell 2010;141:1068–79. 20537373 10.1016/j.cell.2010.04.035

[12] Dai J, Patzke C, Liakath-Ali K. et al. GluD1 is a signal transduction device disguised as an ionotropic receptor. Nature 2021;595:261–5. 34135511 10.1038/s41586-021-03661-6 PMC8776294

[13] Kashiwabuchi N, Ikeda K, Araki K. et al. Impairment of motor coordination, Purkinje cell synapse formation, and cerebellar long-term depression in GluRδ2 mutant mice. Cell 1995;81:245–52. 7736576 10.1016/0092-8674(95)90334-8

[14] Takeo YH, Shuster SA, Jiang L. et al. GluD2- and Cbln1-mediated competitive interactions shape the dendritic arbors of cerebellar Purkinje cells. Neuron 2021;109:629–644.e8. 33352118 10.1016/j.neuron.2020.11.028 PMC8833808

[15] Kurihara H, Hashimoto K, Kano M. et al. Impaired parallel Fiber→Purkinje cell synapse stabilization during cerebellar development of mutant mice lacking the glutamate receptor δ2 subunit. J Neurosci 1997;17:9613–23. 9391016 10.1523/JNEUROSCI.17-24-09613.1997 PMC6573399

[16] Griswold AJ, Ma D, Cukier HN. et al. Evaluation of copy number variations reveals novel candidate genes in autism spectrum disorder-associated pathways. Hum Mol Genet 2012;21:3513–23. 22543975 10.1093/hmg/dds164 PMC3392110

[17] Glessner JT, Wang K, Cai G. et al. Autism genome-wide copy number variation reveals ubiquitin and neuronal genes. Nature 2009;459:569–73. 19404257 10.1038/nature07953 PMC2925224

[18] Nord AS, Roeb W, Dickel DE. et al. Reduced transcript expression of genes affected by inherited and de novo CNVs in autism. Eur J Hum Genet 2011;19:727–31. 21448237 10.1038/ejhg.2011.24 PMC3110052

[19] Fallin MD, Lasseter VK, Avramopoulos D. et al. Bipolar I disorder and schizophrenia: a 440-single-nucleotide polymorphism screen of 64 candidate genes among Ashkenazi Jewish case-parent trios. Am J Hum Genet 2005;77:918–36. 16380905 10.1086/497703 PMC1285177

[20] Edwards AC, Aliev F, Bierut LJ. et al. Genome-wide association study of comorbid depressive syndrome and alcohol dependence. Psychiatr Genet 2012;22:31–41. 22064162 10.1097/YPG.0b013e32834acd07 PMC3241912

[21] Guo SZ, Huang K, Shi YY. et al. A case-control association study between the GRID1 gene and schizophrenia in the Chinese Northern Han population. Schizophr Res 2007;93:385–90. 17490860 10.1016/j.schres.2007.03.007

[22] Greenwood TA, Lazzeroni LC, Murray SS. et al. Analysis of 94 candidate genes and 12 endophenotypes for schizophrenia from the consortium on the genetics of schizophrenia. Am J Psychiatry 2011;168:930–46. 21498463 10.1176/appi.ajp.2011.10050723 PMC3751972

[23] Yadav R, Gupta SC, Hillman BG. et al. Deletion of glutamate delta-1 receptor in mouse leads to aberrant emotional and social behaviors. PLoS One 2012;7:e32969. 22412961 10.1371/journal.pone.0032969 PMC3296759

[24] Nakamoto C, Kawamura M, Nakatsukasa E. et al. GluD1 knockout mice with a pure C57BL/6N background show impaired fear memory, social interaction, and enhanced depressive-like behavior. PLoS One 2020;15:e0229288. 32078638 10.1371/journal.pone.0229288 PMC7032715

[25] Gupta SC, Yadav R, Pavuluri R. et al. Essential role of GluD1 in dendritic spine development and GluN2B to GluN2A NMDAR subunit switch in the cortex and hippocampus reveals ability of GluN2B inhibition in correcting hyperconnectivity. Neuropharmacology 2015;93:274–84. 25721396 10.1016/j.neuropharm.2015.02.013 PMC4410021

[26] Penzes P, Cahill ME, Jones KA. et al. Dendritic spine pathology in neuropsychiatric disorders. Nat Neurosci 2011;14:285–93. 21346746 10.1038/nn.2741 PMC3530413

[27] Utine GE, Haliloglu G, Salanci B. et al. A homozygous deletion in GRID2 causes a human phenotype with cerebellar ataxia and atrophy. J Child Neurol 2013;28:926–32. 23611888 10.1177/0883073813484967

[28] Hills LB, Masri A, Konno K. et al. Deletions in GRID2 lead to a recessive syndrome of cerebellar ataxia and tonic upgaze in humans. Neurology 2013;81:1378–86. 24078737 10.1212/WNL.0b013e3182a841a3 PMC3806907

[29] Maier A, Klopocki E, Horn D. et al. De novopartial deletion inGRID2presenting with complicated spastic paraplegia. Muscle Nerve 2014;49:289–92. 24122788 10.1002/mus.24096

[30] Taghdiri M, Kashef A, Abbassi G. et al. Further delineation of the phenotype caused by a novel large homozygous deletion of GRID2 gene in an adult patient. Clin Case Rep 2019;7:1149–53. 31183084 10.1002/ccr3.2020 PMC6552953

[31] Van Schil K, Meire F, Karlstetter M. et al. Early-onset autosomal recessive cerebellar ataxia associated with retinal dystrophy: new human hotfoot phenotype caused by homozygous GRID2 deletion. Genet Med 2015;17:291–9. 25122145 10.1038/gim.2014.95

[32] Veerapandiyan A, Enner S, Thulasi V. et al. A rare syndrome of GRID2 deletion in 2 siblings. Child Neurol Open 2017;4:2329048X1772616. 10.1177/2329048X17726168 PMC5570108 28856174

[33] Kushima I, Aleksic B, Nakatochi M. et al. Comparative analyses of copy-number variation in autism spectrum disorder and schizophrenia reveal etiological overlap and biological insights. Cell Rep 2018;24:2838–56. 30208311 10.1016/j.celrep.2018.08.022

[34] Kakegawa W, Miyoshi Y, Hamase K. et al. D-serine regulates cerebellar LTD and motor coordination through the δ2 glutamate receptor. Nat Neurosci 2011;14:603–11. 21460832 10.1038/nn.2791

[35] Mandolesi G, Autuori E, Cesa R. et al. GluRdelta2 expression in the mature cerebellum of hotfoot mice promotes parallel fiber synaptogenesis and axonal competition. PLoS One 2009;4:e5243–3. 19370152 10.1371/journal.pone.0005243 PMC2666267

[36] Phillips RJS . ‘Lurcher’, a new gene in linkage group XI of the house mouse. J Genet 1960;57:35–42.

[37] Zuo J, De Jager PL, Takahashi KA. et al. Neurodegeneration in Lurcher mice caused by mutation in delta2 glutamate receptor gene. Nature 1997;388:769–73. 9285588 10.1038/42009

[38] Vogel MW, Caston J, Yuzaki M. et al. The Lurcher mouse: fresh insights from an old mutant. Brain Res 2007;1140:4–18. 16412991 10.1016/j.brainres.2005.11.086

[39] Wollmuth LP, Kuner T, Jatzke C. et al. The Lurcher mutation identifies delta 2 as an AMPA/kainate receptor-like channel that is potentiated by Ca(2+). J Neurosci 2000;20:5973–80. 10934245 10.1523/JNEUROSCI.20-16-05973.2000 PMC6772614

[40] Hansen KB, Naur P, Kurtkaya NL. et al. Modulation of the dimer interface at ionotropic glutamate-like receptor delta2 by D-serine and extracellular calcium. J Neurosci 2009;29:907–17. 19176800 10.1523/JNEUROSCI.4081-08.2009 PMC2806602

[41] Coutelier M, Burglen L, Mundwiller E. et al. GRID2 mutations span from congenital to mild adult-onset cerebellar ataxia. Neurology 2015;84:1751–9. 25841024 10.1212/WNL.0000000000001524

[42] Jones KS, VanDongen HM, VanDongen AM. The NMDA receptor M3 segment is a conserved transduction element coupling ligand binding to channel opening. J Neurosci 2002;22:2044–53. 11896144 10.1523/JNEUROSCI.22-06-02044.2002 PMC6758261

[43] Yuan H, Erreger K, Dravid SM. et al. Conserved structural and functional control of N-methyl-D-aspartate receptor gating by transmembrane domain M3. J Biol Chem 2005;280:29708–16. 15970596 10.1074/jbc.M414215200

[44] Davies B, Brown LA, Cais O. et al. A point mutation in the ion conduction pore of AMPA receptor GRIA3 causes dramatically perturbed sleep patterns as well as intellectual disability. Hum Mol Genet 2017;26:3869–82. 29016847 10.1093/hmg/ddx270 PMC5639461

[45] Geisheker MR, Heymann G, Wang T. et al. Hotspots of missense mutation identify neurodevelopmental disorder genes and functional domains. Nat Neurosci 2017;20:1043–51. 28628100 10.1038/nn.4589 PMC5539915

[46] Hu Z, Xiao X, Zhang Z. et al. Genetic insights and neurobiological implications from NRXN1 in neuropsychiatric disorders. Mol Psychiatry 2019;24:1400–14. 31138894 10.1038/s41380-019-0438-9

[47] Harrison V, Connell L, Hayesmoore J. et al. Compound heterozygous deletion of NRXN1 causing severe developmental delay with early onset epilepsy in two sisters. Am J Med Genet A 2011;155a:2826–31. 21964664 10.1002/ajmg.a.34255

[48] Clarke RA, Lee S, Eapen V. Pathogenetic model for Tourette syndrome delineates overlap with related neurodevelopmental disorders including autism. Transl Psychiatry 2012;2:e158. 22948383 10.1038/tp.2012.75 PMC3565204

[49] Seigneur E, Wang J, Dai J. et al. Cerebellin-2 regulates a serotonergic dorsal raphe circuit that controls compulsive behaviors. Mol Psychiatry 2021;26:7509–21. 34158618 10.1038/s41380-021-01187-x PMC8692491

[50] Shibata M, Pattabiraman K, Muchnik SK. et al. Hominini-specific regulation of CBLN2 increases prefrontal spinogenesis. Nature 2021;598:489–94. 34599306 10.1038/s41586-021-03952-y PMC9018127

[51] Traynelis J, Silk M, Wang Q. et al. Optimizing genomic medicine in epilepsy through a gene-customized approach to missense variant interpretation. Genome Res 2017;27:1715–29. 28864458 10.1101/gr.226589.117 PMC5630035

[52] Perszyk RE, Kristensen AS, Lyuboslavsky P. et al. Three-dimensional missense tolerance ratio analysis. Genome Res 2021;31:1447–61. 34301626 10.1101/gr.275528.121 PMC8327912

[53] Matsuda K, Miura E, Miyazaki T. et al. Cbln1 is a ligand for an orphan glutamate receptor delta2, a bidirectional synapse organizer. Science 2010;328:363–8. 20395510 10.1126/science.1185152

[54] Armstrong CL, Duffin CA, McFarland R. et al. Mechanisms of compartmental purkinje cell death and survival in the lurcher mutant mouse. Cerebellum 2011;10:504–14. 21104177 10.1007/s12311-010-0231-4

[55] Yadav R, Rimerman R, Scofield MA. et al. Mutations in the transmembrane domain M3 generate spontaneously open orphan glutamate delta1 receptor. Brain Res 2011;1382:1–8. 21215726 10.1016/j.brainres.2010.12.086

[56] Ali Z, Zulfiqar S, Klar J. et al. Homozygous GRID2 missense mutation predicts a shift in the D-serine binding domain of GluD2 in a case with generalized brain atrophy and unusual clinical features. BMC Med Genet 2017;18:144. 29207948 10.1186/s12881-017-0504-6 PMC5718074

[57] Chin AC, Yovanno RA, Wied TJ. et al. D-serine potently drives ligand-binding domain closure in the ionotropic glutamate receptor GluD2. Structure 2020;28:1168–1178.e2. 32735769 10.1016/j.str.2020.07.005 PMC7544663

[58] Kristensen AS, Hansen KB, Naur P. et al. Pharmacology and structural analysis of ligand binding to the orthosteric site of glutamate-like GluD2 receptors. Mol Pharmacol 2016;89:253–62. 26661043 10.1124/mol.115.100909 PMC4746485

[59] Dravid SM, Erreger K, Yuan H. et al. Subunit-specific mechanisms and proton sensitivity of NMDA receptor channel block. J Physiol 2007;581:107–28. 17303642 10.1113/jphysiol.2006.124958 PMC2075223

[60] Williams K, Dattilo M, Sabado TN. et al. Pharmacology of delta2 glutamate receptors: effects of pentamidine and protons. J Pharmacol Exp Ther 2003;305:740–8. 12606689 10.1124/jpet.102.045799

[61] Jeromin A, Huganir RL, Linden DJ. Suppression of the glutamate receptor delta 2 subunit produces a specific impairment in cerebellar long-term depression. J Neurophysiol 1996;76:3578–83. 8930298 10.1152/jn.1996.76.5.3578

[62] Nakagami R, Kohda K, Kakegawa W. et al. Phosphorylation of delta2 glutamate receptors at serine 945 is not required for cerebellar long-term depression. Keio J Med 2008;57:105–10. 18677091 10.2302/kjm.57.105

[63] Burada AP, Vinnakota R, Kumar J. Cryo-EM structures of the ionotropic glutamate receptor GluD1 reveal a non-swapped architecture. Nat Struct Mol Biol 2020;27:84–91. 31925409 10.1038/s41594-019-0359-y PMC7025878

[64] Burada AP, Vinnakota R, Kumar J. The architecture of GluD2 ionotropic delta glutamate receptor elucidated by cryo-EM. J Struct Biol 2020;211:107546. 32512155 10.1016/j.jsb.2020.107546

[65] Gantz SC, Moussawi K, Hake HS. Delta glutamate receptor conductance drives excitation of mouse dorsal raphe neurons. elife 2020;9:e56054. 10.7554/eLife.56054 PMC7180053 32234214

[66] Schmid SM, Hollmann M. To gate or not to gate: are the delta subunits in the glutamate receptor family functional ion channels? Mol Neurobiol 2008;37:126–41. 18521762 10.1007/s12035-008-8025-0

[67] Schmid SM, Kott S, Sager C. et al. The glutamate receptor subunit delta2 is capable of gating its intrinsic ion channel as revealed by ligand binding domain transplantation. Proc Natl Acad Sci U S A 2009;106:10320–5. 19506248 10.1073/pnas.0900329106 PMC2700928

[68] XiangWei W, Kannan V, Xu Y. et al. Heterogeneous clinical and functional features of GRIN2D-related developmental and epileptic encephalopathy. Brain 2019;142:3009–27. 31504254 10.1093/brain/awz232 PMC6763743

[69] Yuan H, Hansen KB, Zhang J. et al. Functional analysis of a de novo GRIN2A missense mutation associated with early-onset epileptic encephalopathy. Nat Commun 2014;5:3251. 24504326 10.1038/ncomms4251 PMC3934797

[70] Hansen KB, Tajima N, Risgaard R. et al. Structural determinants of agonist efficacy at the glutamate binding site of N-methyl-D-aspartate receptors. Mol Pharmacol 2013;84:114–27. 23625947 10.1124/mol.113.085803 PMC3684824

[71] Swanger SA, Chen W, Wells G. et al. Mechanistic insight into NMDA receptor dysregulation by rare variants in the GluN2A and GluN2B agonist binding domains. Am J Hum Genet 2016;99:1261–80. 27839871 10.1016/j.ajhg.2016.10.002 PMC5142120

[72] Zhong C, Shen J, Zhang H. et al. Cbln1 and Cbln4 are structurally similar but differ in GluD2 binding interactions. Cell Rep 2017;20:2328–40. 28877468 10.1016/j.celrep.2017.08.031

[73] Sali A, Blundell TL. Comparative protein modelling by satisfaction of spatial restraints. J Mol Biol 1993;234:779–815. 8254673 10.1006/jmbi.1993.1626

[74] Shen MY, Sali A. Statistical potential for assessment and prediction of protein structures. Protein Sci 2006;15:2507–24. 17075131 10.1110/ps.062416606 PMC2242414

[75] Twomey EC, Yelshanskaya MV, Vassilevski AA. et al. Mechanisms of channel block in calcium-permeable AMPA receptors. Neuron 2018;99:956–968.e4. 30122377 10.1016/j.neuron.2018.07.027 PMC6181147

[76] Rook ML, Miaro M, Couch T. et al. Mutation of a conserved glutamine residue does not abolish desensitization of acid-sensing ion channel 1. J Gen Physiol 2021;153:e202012855. 10.1085/jgp.202012855 PMC8167889 34061161

[77] Ran FA, Hsu PD, Wright J. et al. Genome engineering using the CRISPR-Cas9 system. Nat Protoc 2013;8:2281–308. 24157548 10.1038/nprot.2013.143 PMC3969860

[78] Elegheert J, Behiels E, Bishop B. et al. Lentiviral transduction of mammalian cells for fast, scalable and high-level production of soluble and membrane proteins. Nat Protoc 2018;13:2991–3017. 30455477 10.1038/s41596-018-0075-9 PMC6364805

[79] Chen Y, Liu B, Ju L. et al. Fluorescence biomembrane force probe: concurrent quantitation of receptor-ligand kinetics and binding-induced intracellular signaling on a single cell. J Vis Exp 2015;102:e52975 in press. 10.3791/52975 PMC4544851 26274371

[80] Chen W, Evans EA, McEver RP. et al. Monitoring receptor-ligand interactions between surfaces by thermal fluctuations. Biophys J 2008;94:694–701. 17890399 10.1529/biophysj.107.117895 PMC2157231

[81] Perszyk RE, Swanger SA, Shelley C. et al. Biased modulators of NMDA receptors control channel opening and ion selectivity. Nat Chem Biol 2020;16:188–96. 31959964 10.1038/s41589-019-0449-5 PMC6986806

