# Supplementary material for: Clinical features, functional consequences, and rescue pharmacology of missense GRID1 and GRID2 human variants
Source: Hum Mol Genet. 2023 Nov 7;33(4):355–73. doi: 10.1093/hmg/ddad188 (PMC10840383; doi:10.1093/hmg/ddad188)
Supplement: GRID_Variants_Supplemental_Revised_10-26-2023_FINAL_Rev_ddad188 [file grid_variants_supplemental_revised_10-26-2023_final_rev_ddad188.pdf]

SUPPLEMENTAL MATERIAL

Clinical features, functional consequences, and rescue pharmacology of missense *GRID1* and *GRID2* human variants

James P. Allen[1,2,§], Kathryn B. Garber[3,4,§], Riley Perszyk[1], Cara T. Khayat[5], Steven A. Kell[1,6], Maki Kaneko[7,8], Catherine Quindipan[8], Sulagna Saitta[9], Roger L. Ladda[10], Stacy Hewson[11], Michal Inbar-Feigenberg[12], Chitra Prasad[13], Asuri N. Prasad[14], Leah Olewiler[15], Weiyi Mu[16], Liana S. Rosenthal[17], Marcello Scala[18,19], Pasquale Striano[18,19], Federico Zara[20], Tyler W McCullock[21], Robin-Tobias Jauss[22], Johannes R. Lemke[22], David M. MacLean[21], Cheng Zhu[5], Hongjie Yuan[1], Scott J. Myers[1,2], Stephen F. Traynelis[1,2*]

[1] Dept. of Pharmacology and Chemical Biology, Emory University School of Medicine, Atlanta, GA 30322.
[2] Center for the Functional Evaluation of Rare Variants (CFERV), Emory University School of Medicine, Atlanta, GA 30322.
[3] Dept. of Human Genetics, Emory University School of Medicine, Atlanta GA 30322
[4] EGL Genetics, Tucker, GA 30084
[5] Dept. of Biomedical Engineering, Georgia Institute of Technology, Atlanta GA 30332
[6] Dept. of Chemistry, Emory University School of Medicine, Atlanta, GA 30322.
[7] Division of Genomic Medicine, Department of Pathology, Children's Hospital Los Angeles, Los Angeles, CA 90027.
[8] Center for Personalized Medicine, Children's Hospital Los Angeles, Los Angeles, CA 90027.
[9] Division of Clinical Genetics, Departments of Human Genetics, OBGYN and Pediatrics, David Geffen School of Medicine at UCLA, Los Angeles, CA 90095
[10] Division of Human Genetics, Department of Pediatrics, Penn State College of Medicine, Hershey, PA 17033
[11] Dept. of Genetic Counselling, The Hospital for Sick Children and Department of Molecular Genetics, University of Toronto, Toronto, ON, Canada M5G 1X8
[12] Division of Clinical & Metabolic Genetics, The Hospital for Sick Children and Pediatrics, University of Toronto, Toronto ON, Canada M5G 1X8
[13] Dept. of Pediatrics (Section of Genetics and Metabolism), Western University and Schulich School of Medicine and Dentistry, Children's Hospital LHSC, London, ON, Canada. N6A5W9
[14] Division of Pediatric Neurology, Department of Pediatrics and Clinical Neurological Sciences, Western University and Schulich School of Medicine and Dentistry, Children's Hospital LHSC, London, ON, Canada. N6A5W9
[15] Dept. of Pediatrics, Division of Medical Genetics, University of Mississippi Medical Center, Jackson, MS 39216
[16] Dept. of Genetic Medicine, Johns Hopkins University, Baltimore MD 21287
[17] Dept. of Neurology, Johns Hopkins University, Baltimore MD 21287
[18] Department of Neurosciences, Rehabilitation, Ophthalmology, Genetics, Maternal and Child Health, Università Degli Studi di Genova, Genoa, Italy
[19] Pediatric Neurology and Muscular Diseases Unit, IRCCS Istituto Giannina Gaslini, Genoa, Italy
[20] Medical Genetics Unit, IRCCS Istituto Giannina Gaslini, Genoa, Italy
[21] Dept. Pharmacology and Physiology, University of Rochester Medical Center, Rochester NY, 14642
[22] Institute of Human Genetics, University of Leipzig Medical Center, 04103 Leipzig, Germany.

§ Contributed equally to this work
* Corresponding author

Table of Contents

Supplemental Table S1. Gene-level tolerance to variation of *GRID1* and *GRID2* compared to other glutamate receptors

Supplemental Table S2: *GRID1* variants collected for this study and from the literature

Supplemental Table S3: *GRID2* Variants collected in this study and from the literature

Supplemental Table S4: *GRID1* missense variants in ClinVar

Supplemental Table S5: *GRID2* missense variants in ClinVar

Supplemental Table S6. Predicted percent occupancy of GluD1-Cbln2 salt bridge interactions

Supplemental Table S7. Predicted percent occupancy of GluD1-Cbln2 hydrogen bond interactions

Supplemental Table S8. Inhibitors of constitutively active GluD2 current

Supplemental Figure S1. Alignment of human and rat GluD1 and GluD2 protein sequences

Supplemental Figure S2. RMSD of GluD1-Cbln2 homology model across MD simulation frames

Supplemental Figure S3. Genotype of the GluD1 KO cell line.

Supplemental Figure S4. 3D MTR analysis of other *GRID1* and *GRID2* variants

Supplemental Figure S5. Flow cytometry validation of GluD1 and Cbln2 presence**.**

Supplemental Figure S6. Single-concentration screen of compounds on GluD2-T649A and GluD2-A654T in oocytes

Supplemental Figure S7. Allosteric interactions of D-serine and Ca^{2+} in GluD2-T649A.

Supplemental Data 1. Models of GluD1, GluD2, and Cbln2

Supplemental Data 2. 3D MTR results of *GRID1*, *GRID2,* and the *GRID1*/*CBLN2* interaction

Supplemental Table S1. Gene-level tolerance to variation of *GRID1* and *GRID2* compared to other glutamate receptors

Gene	RVIS Score	RVIS Percentile
GRIN2B	-2.7495	1.28%
GRIN2A	-2.404	1.96%
GRIA1	-2.1651	2.55%
GRID1	**-1.7687**	**4.21%**
GRIA4	-1.7162	4.57%
GRIK3	-1.6626	4.91%
GRIA3	-1.4335	5.49%
GRIN1	-1.4048	6.92%
GRIK2	-1.3401	7.78%
GRID2	**-1.2628**	**8.77%**
GRIA2	-1.1199	10.77%
GRIN2D	-1.0728	11.66%
GRIK4	-1.0141	12.96%
GRIK1	-0.9506	14.26%
GRIK5	-0.6905	21.25%
GRIN2C	0.2666	61.98%
GRIN3A	0.3998	67.64%
GRIN3B	2.7801	98.85%

Residual Variation Intolerance Score (RVIS) scores for gene level intolerance (Petrovski et al., 2013) are presented. Lower scores reflect less tolerance to variation. *GRID1* and *GRID2* are within the 4.21% and 8.77% of RVIS scores for all genes. This indicates that these genes are highly intolerant to variation. These scores are close to other well-studied ionotropic glutamate receptors that have been implicated in neurological diseases such as *GRIN1*, *GRIN2A*, *GRIN2B*, and *GRIN2D*. All genes represented are members of the ionotropic glutamate receptor family (S. F. Traynelis et al., 2010). RVIS scores are based on gnomAD (ExAC v2) release 2.0 (accessed: March 15th 2017).

Supplemental Table S2: *GRID1* variants collected for this study and from the literature

Nucleotide Change*	Protein Change	Domain	SIFT	PolyPhen	gnomAD AC	# of people	Zygosity	Inheritance	Phenotype	Source
c.220C>T	p.Gln74*	NTD	NA	NA	0	2	2 Heterozygous	Maternal	Pt. 1: Motor delays, intellectual disability, macrocephaly, hypotonia	This study
c.353G>A	p.Arg118His	NTD	deleterious low confidence	probably damaging	2	1	1 Heterozygous	Unknown	Seizure panel ordered. No specific clinical information provided.	This study
c.359C>A	p.Pro120Gln	NTD	Tolerated	benign	0	7	7 Heterozygous	Pt 1, 3-6: Unknown, Pt 2: Maternal	Pt 1: Autism, Pt 2: Abnormal newborn screen, Pt. 3: Hereditary hemorrhagic telangiectasia, Pt. 4-6: Cholestasis	This study
c.379G>A	p.Ala127Thr	NTD	Tolerated	benign	23	8	8 Heterozygous	Pt. 1, 3-6: Unknown, Pt 2: Maternal	Pt. 1: Hypoplasia of cerebellar vermis, thinning of corpus callosum, hypotonia, motor and developmental delays, Pt. 2: Seizures, developmental delay, nystagmus, hypotonia, cardiac malformation, Pt. 3: Hemihypertrophy and abnormal pigmentation, Pt. 4: Hearing impairment, autism, Pt. 5: Usher syndrome, Pt. 6-7: Cholestasis	This study
c.407G>A	p.Gly136Asp	NTD	tolerated	benign	14	8	8 Heterozygotes	Pt 1: Unknown, Pt. 2: Paternal Pt. 3-7: Unknown	Pt. 1: Optic atrophy, Pt. 2: developmental delay, hypotonia, seizures, brain abnormalities, cardiac malformation, Pt. 3: Hypermobiliy, joint dislocations, joint pain, Pt. 3: Wilson disease. Alternate molecular diagnosis. Pt. 5-7: Cholestasis	This study
c.437C>T; c.442C>T	p.Pro146Leu; p.Arg148Cys	NTD	Deleterious; tolerated	Probably damaging; probably damaging	0;3	1	Compound heterozygous	Maternal (c.437C>T); Paternal (c.442C>T)	Abnormality of neuronal migration/Malformation of cortical development. Parents with single heterozygous variants are healthy.	This study
c.481C>T	p.Arg161Cys	NTD	deleterious	benign	0	3	1 Homozygous, 2 Heterozygous	Pt. 1: 1allele paternal; mother not tested Pt. 2-3: Unknown	Pt. 1: Homozygote with scoliosis, hip problems joint pain. Alternate molecular diagnosis for presenting features. Pt. 2: Heterozygote with syndactyly, short stature. Pt. 3: heterozygote with cholestasis	This study
c.482G>A	p.Arg161His	NTD	tolerated low confidence	benign	4	6	3 Homozygous 3 Heterozygous	Homozygotes inherited variant from each heterozygous parent	Pt. 1: Homozygote with mild intellectual disability, spastic paraplegia, glaucoma, optic atrophy, Pt. 2: Homozygote with mild intellectual disability, spastic paraplegia, glaucoma, optic atrophy, Pt. 3: Homozygote with moderate intellectual disability, spastic paraplegia, glaucoma, blindness; Heterozygous parents and sibling unaffected	Ung et al. 2022
c.496G>A	p.Val166Ile	NTD	tolerated	benign	86	18	Unknown	Unknown	Pt. 1: Intellectual disability, Pt. 2: Learning disorder, ADHD, anxiety, Pt. 3: Autism	This study
c.499A>G	p.Met167Val	NTD	tolerated	benign	9	2	2 Heterozygous	Unknown	Clinical samples with unknown referring phenotype	This study
c.596A>G	p.Lys199Arg	NTD	tolerated	benign	0	1	1 Heterozygous	Unknown	Seizure-like activity and possible ataxia	This study
c.662G>A	p.Arg221Gln	NTD	tolerated	probably damaging	7	5	5 Heterozygous	Unknown	Pt. 1: Intellectual disability, Pt. 2: Developmental delay, Pt. 3: Inflammatory Bowel Disease, Pt. 4: Epilepsy, Pt. 5: Hemihypertrophy, capillary malformations	This study
c.706G>T	p.Ala236Ser	NTD	deleterious	probably damaging	0	1	1 Heterozygous	Unknown	Focal seizures	This study
c.857C>T	p.Pro286Leu	NTD	tolerated	probably damaging	7	1	1 Heterozygous	Unknown	Developmental delays, speech delay. This patient had a likely pathogenic variant in a different gene that is diagnostic.	This study
c.899G>T	p.Arg300Leu	NTD	deleterious	probably damaging	0	1	1 Heterozygous	Unknown	Intellectual disability	This study
c.919G>A	p.Asp307Asn	NTD	tolerated	probably damaging	5	1	1 Heterozygous	Unknown	Epileptic encephalopathy, absence seizures, tonic-clonic seizures, ataxia	This study
c.1022G>A	p.Arg341Gln	NTD	tolerated	probably damaging	0	1	Homozygous	Unknown	obesity with insatiable appetite, mild to moderate intellectual disability, ADHD, marked aggression, anxiety, schizoaffective disorder, and parents with intellectual disability. Hypotonia in the newborn period and early infancy. Facial dysmorphism, congenital v shape scar, chubby face, extensive stretch marks and large hands and feet.	This study
c.1119C>A	p.His373Gln	NTD	tolerated	benign	2	1	1 Heterozygous	Unknown	Epilepsy. Alternate molecular diagnosis.	This study
c.1151G>A	p.Arg384Gln	NTD	tolerated	benign	34	4	4 Heterozygous	Unknown	Pt. 1: Optic atrophy, Pt. 2: Intellectual disability, spastic paraplegia, Pt. 3: Autism. Alternate molecular diagnosis, Pt. 4: Skeletal dysplasia with increased bone density	This study
c.1285C>A	p.Pro429Thr	NTD-S1	tolerated	benign	8	1	Homozygous	Unknown	Dev delay, febrile illness, hypotonia, feeding difficulties, abnormal MRI, breathing difficulties	This study

c.1339G>A	p.Glu447Lys	S1	deleterious	benign	0	1	1 Heterozygous	Unknown	Myoclonic epilepsy	This study
c.1348G>A	p.Val450Met	S1	deleterious	possibly damaging	13	7	7 Heterozygous	Pt 1: Paternal, Pt 2-6: Unknown	Pt. 1: Autism, language and fine motor regression. Not found in similarly affected sibling, Pt. 2: Osteogenesis imperfecta, Pt. 3: Pectus excavatum and hyperflexibility, Pt. 4: Cholestasis. Had heterozygous Likely Pathogenic variant in a relevant gene with autosomal recessive inheritance., Pt. 5: Cholestasis, Pt. 6 Wilson disease	This study
c.1366A>C ; c.2255C>T	p.Ile456Leu; p.Thr752Met	S1; S2	tolerated, deleterious	benign; uncertain	0; 11	2;3	1 Compound Heterozygous, 1 Heterozygous	Maternal (I456L) and paternal (T752M); Unknown	Pt. 1: Autism, developmental delay, microcephaly, abnormal brain MRI. Parents with single variants are healthy	This study
c.1808C>A	p.Ala603Asp	M1-M3	deleterious	benign	32	3	3 Heterozygous	Pt. 1: Unknown, Pt. 2: Paternal	2 probands, 1 parent. Pt. 1: Immunodeficiency; Alternate molecular diagnosis. Pt. 2: Global development delay, spastic quadriparesis, seizures, macrocephaly. Has Likely Pathogenic de novo variant in a different gene that is diagnostic.	This study
c.1811C>T	p.Thr604Ile	M1-M3	deleterious	possibly damaging	20	5	4 Heterozygous, 1 unknown	Unknown	Pt. 1: Schizencephaly and septo-optic dysplasia, Pt. 2: Motor delays, hypertonia, dystonic encephalopathy, Pt. 3:Generalized epilepsy, neonatal seizure, Pt. 4: Pierre Robin sequence, bulbar neurologic losses, Pt. 5: Severe hypotonia, inverted nipples, respiratory failure	This study
c.1948G>A	p.Ala650Thr	M3	deleterious	probably damaging	1	Unknown	Heterozygous	Unknown	Schizophrenia	SCHEMA
c.2050G>C	p.Asp684His	S2	deleterious	possibly damaging	0	1	Homozygous	Unknown	recurrent bilateral otitis media, reactive airway disease, bilateral vesicoureteral reflux, hydronephrosis, unilateral atrophic kidney, left club foot and left arm amelia, stereotypies, developmentally appropriate	This study
c.2120A>G	p.Glu707Gly	S2	deleterious	probably damaging	0	1	Heterozygous	Unknown	Epilepsy	This study
c.2146G>A	p.Gly716Arg	S2	tolerated	benign	10	1	Heterozygous	Unknown	Developmental delay, seizures	This study
c.2449G>A	p.Ala817Thr	S2-M4	tolerated	benign	0	1	Heterozygous	*de novo*	Developmental delay, intellectual disability, abnormal behavior, neuropsychiatric features	This study
c.2690C>T	p.Ser897Leu	CTD	tolerated	probably damaging	1	2	Heterozygous	Paternal	Epilepsy, ADHD, learning disabilities, macrocephaly. Father also has epilepsy. Alternate molecular diagnosis segregated with epilepsy.	This study
c.2855C>T	p.Pro952Leu	CTD	tolerated	probably damaging	23	5	5 Heterozygous	Unknown	Pt. 1: Developmental delays, ADHD, and dysmorphic features, Pt. 2: Short stature, ptosis, pectus excavatum, developmental delays, small ears, Pt. 3: Parent of tested individual. Variant not in child., Pt. 4: Cholestasis, Pt. 5: Short limbs, hepatosplenomegaly, bone density abnormalities, dysmorphic features, atrophy of the corpus callosum, Pt. 2: Eye panel was ordered, no specific clinical info provided. 1 heterozygous pathogenic variant was found in a different gene with autosomal recessive inheritance.	This study
c.2950C>T	p.Pro984Ser	CTD	tolerated	benign	31	2	Heterozygous	Unknown	Pt. 1: Epilepsy, Pt. 2: Cholestasis, Alternate molecular diagnosis	This study

Supplemental Table S3: *GRID2* Variants collected in this study and from the literature

Nucleotide Change*	Protein Consequence	Domain	SIFT	PolyPhen	gnomAD AC	# of people	Zygosity	Inheritance	Phenotype	Source
c.52T>C	p.Trp18Arg	SigPep	tolerated	benign	0	1	Heterozygous	Inherited	Ataxia. Proband has variants in *SPTBN2 and WFS1*	Fogel et al., 2014
c.210delT	p.Asp71Met*fs**16	NTD	NA	NA	0	1	Heterozygous	*de novo*	congenital hypothyroidism, hypotonia, joint hypermobility, developmental delay (speech and fine motor), conductive hearing loss, macrocephaly, clinodactyly, and distinctive facial features	Personal commun. §
c.899G>A	p.Arg300His	NTD	tolerated	possibly damaging	9	1	Heterozygous	Unknown	adult-onset progressive cerebellar ataxia, parkinsonism, diplopia, dysphagia	This study
c.1945A>G	p.Thr649Ala	M3	deleterious	probably damaging	0	1	Heterozygous	*de novo*	cerebellar ataxia, dysarthria, strabismus, and significant cerebellar volume loss on MRI	This study
c.1960G>A	p.Ala654Thr	M3	deleterious	probably damaging	0	1	Heterozygous	*de novo*	Congenital spinocerebellar ataxia, saccadic pursuit, nystagmus, cerebellar atrophy	Coutelier et al 2015
c.1961C>A	p.Ala654Asp	M3	deleterious	probably damaging	0	1	Heterozygous	*de novo*	Congenital Spinocerebellar ataxia, saccadic pursuit, nystagmus, cerebellar atrophy	Coutelier et al., 2015
c.1966C>G	p.Leu656Val	M3-S2	deleterious	probably damaging	0	6	Homozygous, Heterozygous	Semi-dominant	slowly progressive cerebellar ataxia or congenital ataxia, Intellectual disability, occularmotor symptoms	Coutelier et al., 2015
c.2128C>T	p.Arg710Trp	S2	deleterious	possibly damaging	13	3	Homozygous	Inherited	Cerebellar atrophy, cognitive impairment, muscular hypotonia, poor speech,spinocerebellar ataxia; autosomal recessive 18	Ali et al., 2017
c.2921T>A	p.Phe974Tyr	CTD	deleterious low confidence	possibly damaging	304	2	Heterozygous	Maternal	Proband and parent. microcephaly, hypotonia, motor and speech delay, dysmorphic features, spastic gait. Had alternate molecular diagnosis.	This study
GRCh38: chr4:92930845-93092974	Copy number variant GRID2 deletion	-	NA	NA	0	1	Heterozygous	Unknown	Schizophrenia, autistic traits	Kushima et al., 2018
GRCh38: chr4:92590068-92732357	Copy number variants GRID2 deletion	-	NA	NA	0	1	Heterozygous	Unknown	Schizophrenia	Kushima et al., 2018
GRCh37: chr4:92981313-93256907	276 kb deletion spanning exon 1	-	NA	NA	0	1	Heterozygous	*de novo*	Cerebellar ataxia, spastic paraplegia, frontotemporal dementia	Maier et al., 2014
GRCh37: chr4:93422866_93754032	p.Gly30_Glu81del In-frame deletion exon 2	-	NA	NA	0	1	Homozygous	Inherited	Cerebellar ataxia, hypotonia nystagmus	Van Schil et al., 2015
GRCh37:~chr4:93525000-94836000	1.3 Mb deletion on 4q22.1q22.2 (exons 3-16)	-	NA	NA	0	2	Homozygous	Inherited	Movement disorders, ataxia, developmental delay	Taghdiri et al., 2019
GRCh37:chr4:93412943-93748082 (de novo), chr4:93481110-93531257	Deletion of exon 2	-	NA	NA	0	1	Compound heterozygous	Inherited / *de novo*	Ataxia, tonic upgaze, nystagmus, developmental delay	Hills et al., 2014
GRCh37: chr4:94019842-94056765	Deletion of exon 4	-	NA	NA	0	3	Homozygous	Inherited	Ataxia, tonic upgaze, nystagmus, developmental delay	Hills et al., 2014
GRCh37: chr4:94153589-94298037	Deletions of exon 3 and 4	-	NA	NA	0	3	Homozygous	Inherited	Nystagmus, hypotonia, movement disorders, cerebellar ataxia, developmental delay	Utine et al., 2013
GRCh37: chr4:93472963-93639305	166-kb homozygous intragenic deletion at 4q22.1 →22.1 (exon 2)	-	NA	NA	0	2	Homozygous	Inherited	Ataxia, Tonic upgaze, nystagmus, hypotonia, developmental delay	Veerapandiyan et al., 2017
GRCh37: Chr4:94316480-94471802-	Deletion at 4q22.2 (Exon 9-13)	-	NA	NA	0	3	Heterozygous	Inherited	Cerebellar atrophy and ataxia, nystagmus, hypotonia	This study
GRCh37: Chr4:94137618-94159909	Deletion 4q22.2 (exon 6-8)	-	NA	NA	0	3	Heterozygous	Inherited	Cerebellar atrophy and ataxia, nystagmus, hypotonia	This study

§ Personal Communication, Dr. Juvianee I. Estrada-Veras

Supplemental Table S4: *GRID1* missense variants in ClinVar

Nucleotide Change*	Protein Change	Domain	SIFT	PolyPhen	gnomAD	Phenotype	Accession
c.349C>G	p.Gln117Glu	NTD	deleterious low confidence	possibly damaging	0	Inborn genetic diseases	VCV002559081
c.379G>A	p.Ala127Thr	NTD	tolerated	possibly damaging	23	Inborn genetic diseases	VCV002347288
c.437C>T	p.Pro146Leu	NTD	deleterious	probably damaging	0	Malformation of cortical development, inherited (maternal)	VCV000208924
c.442C>T	p.Arg148Cys	NTD	tolerated	probably damaging	3	Malformation of cortical development, inherited (paternal)	VCV000208923
c.482G>A	p.Arg161His	NTD	tolerated	benign	4	Inborn genetic diseases	VCV002408589
c.662G>A	p.Arg221Gln	NTD	tolerated	probably damaging	7	Inborn genetic diseases	VCV002389797
c.673C>T	p.Arg225Cys	NTD	deleterious	probably damaging	2	Inborn genetic diseases	VCV002265245
c.836C>T	p.Thr279Ile	NTD	tolerated	probably damaging	1	Inborn genetic diseases	VCV002260749
c.1151G>A	p.Arg384Gln	NTD	tolerated	possibly damaging	34	Inborn genetic diseases	VCV002341592
c.1288A>G	p.Met430Val	S1	tolerated	benign	0	Unknown	VCV001279125
c.1496C>A	p.Thr499Asn	S1	tolerated	benign	2	Inborn genetic diseases	VCV002276933
c.1585G>A	p.Val529Ile	S1	deleterious	probably damaging	6319	Unknown	VCV001249619
c.1625G>C	p.Gly542Ala	S1	tolerated	probably damaging	5	Inborn genetic diseases	VCV002295122
c.1639A>G	p.Lys547Glu	S1-M1	deleterious	possibly damaging	4	Inborn genetic diseases	VCV002232535
c.1642C>T	p.Pro548Ser	S1-M1	deleterious	benign	13	Inborn genetic diseases	VCV002309911
c.1808C>A	p.Ala603Asp	M1-M3	tolerated	benign	32	Inborn genetic diseases	VCV002385716
c.1808C>T	p.Ala603Val	M1-M3	tolerated	benign	8	Inborn genetic diseases	VCV002395011
c.1873G>A	p.Val625Met	M1-M3	tolerated	benign	10	Inborn genetic diseases	VCV002211380
c.1911G>T	p.Trp637Cys	M3	deleterious	probably damaging	2	Inborn genetic diseases	VCV002398275
c.2146G>A	p.Gly716Arg	S2	tolerated	benign	10	Inborn genetic diseases	VCV002410007
c.2326C>T	p.His776Tyr	S2	tolerated	probably damaging	0	Inborn genetic diseases	VCV002340170
c.2575C>T	p.Arg859Trp	CTD	deleterious	probably damaging	8	Inborn genetic diseases	VCV002366264
c.2597A>G	p.Lys866Arg	CTD	tolerated	benign	0	Inborn genetic diseases	VCV002308905
c.2984G>A	p.Gly995Asp	CTD	tolerated	probably damaging	0	Inborn genetic diseases	VCV002207898

Missense variants are named on the transcript NM_017551.3; only inherited or germline variants included. Missense variants that are absent from gnomAD are red. ClinVar accessed 9-15-23.

Supplemental Table S5. *GRID2* missense variants in ClinVar

Nucleotide Change*	Protein Consequence	Domain	SIFT	PolyPhen	gnomAD	Phenotype	Accession
c.62C>A	p.Ala21Glu	Signal peptide	tolerated	benign	0	Inborn genetic disease	VCV002278308
c.62C>T	p.Ala21Val	Signal peptide	tolerated	benign	36	Unknown	VCV001419994.2
c.96T>G	p.Ile32Met	NTD	deleterious	probably damaging	3	Inborn genetic disease	VCV002209248
c.117G>T	p.Lys39Asn	NTD	tolerated	probably damaging	3	Inborn genetic disease	VCV002262925
c.121G>T	p.Asp41Tyr	NTD	tolerated	possibly damaging	0	Autosomal recessive spinocerebellar ataxia 18	VCV001031990
c.203C>T	p.Thr68Met	NTD	deleterious	probably damaging	9982	Unknown	VCV001321117
c.212A>C	p.Asp71Ala	NTD	tolerated	probably damaging	10	Inborn genetic disease	VCV002387891
c.290T>C	p.Ile97Thr	NTD	tolerated	possibly damaging	333	Unknown	VCV000726497
c.299C>T	p.Thr100Met	NTD	tolerated	benign	19	Inborn genetic disease	VCV001981186
c.331G>A	p.Ala111Thr	NTD	tolerated	probably damaging	3	Unknown	VCV002096167
c.334A>G	p.Met112Val	NTD	deleterious	probably damaging	0	Inborn genetic disease	VCV002237327
c.355A>G	p.Ile119Val	NTD	tolerated	benign	1	Inborn genetic disease	VCV002361170
c.387T>G	p.Ser129Arg	NTD	tolerated	probably damaging	1	Unknown	VCV002007522
c.517G>C	p.Asp173His	NTD	deleterious	probably damaging	0	Inborn genetic disease	VCV002335198
c.529G>A	p.Asp177Asn	NTD	tolerated	probably damaging	9	Unknown	VCV001900250
c.577A>G	p.Met193Val	NTD	tolerated	benign	77	Unknown	VCV002076785
c.587C>A	p.Ala196Glu	NTD	deleterious	possibly damaging	12	Unknown	VCV001331258
c.614A>G	p.Asn205Ser	NTD	tolerated	benign	28	Unknown	VCV002191914
c.661A>C	p.Asn221His	NTD	tolerated	benign	1	Unknown	VCV001925212
c.671G>A	p.Arg224Gln	NTD	tolerated	probably damaging	3	Spinocerebellar ataxia; autosomal recessive 18, homozygous, inherited	VCV000488523
c.686G>A	p.Arg229Gln	NTD	tolerated	probably damaging	1	Inborn genetic disease	VCV002221805
c.799G>A	p.Asp267Asn	NTD	deleterious	probably damaging	7	Inborn genetic disease	VCV002388002
c.806A>T	p.Asp269Val	NTD	deleterious	probably damaging	2	Unknown	VCV001935299
c.876G>C	p.Gln292His	NTD	deleterious	benign	9	Developmental delays, seizures, white matter abnormalities, microcephaly, truncal hypotonia, hypertonia of extremities, heterozygous	VCV000500424
c.899G>A	p.Arg300His	NTD	deleterious	probably damaging	9	Unknown	VCV000430178
c.994C>T	p.Leu332Phe	NTD	tolerated	probably damaging	2	Inborn genetic disease	VCV002226159
c.1099C>T	p.Arg367Cys	NTD	deleterious	probably damaging	1	Unknown	VCV001932578
c.1107G>A	p.Met369Ile	NTD	tolerated	possibly damaging	0	Unknown	VCV002094811
c.1178A>G	p.Asn393Ser	NTD	deleterious	probably damaging	1	not provided	VCV000426507
c.1241G>A	p.Arg414Gln	NTD	deleterious	probably damaging	19	Unknown	VCV001930621
c.1331G>A	p.Arg444His	S1	tolerated	probably damaging	4	Unknown	VCV001897094
c.1419T>A	p.Asp473Glu	S1	deleterious	probably damaging	10	Child onset for dermatologic,endocrine, musculoskeletal,structural,neurologic Inborn genetic diseases/MR/ID/DD/Movement disorders, overgrowth,hypotonia, heterozygous	VCV000522090
c.1468G>A	p.Val490Ile	S1	tolerated	benign	23682	Unknown	VCV001267971
c.1637G>C	p.Gly546Ala	S1	tolerated	probably damaging	0	Unknown	VCV002027227

c.1658A>G	p.Glu553Gly	S1-M1	tolerated	probably damaging	1	Autosomal recessive spinocerebellar ataxia 18, inherited (maternal)	VCV001027933
c.1690C>T	p.Pro564Ser	S1-M1	deleterious	probably damaging	0	Unknown	VCV001363817
c.1720G>T	p.Ala574Ser	M1	tolerated	possibly damaging	1	Unknown	VCV001985697
c.1775C>T	p.Pro592Leu	M1-M3	deleterious	possibly damaging	33	Inborn genetic disease	VCV002351263
c.1861G>A	p.Gly621Arg	M1-M3	tolerated	probably damaging	10	Inborn genetic disease	VCV002401795
c.1936T>C	p.Ser646Pro	M3	deleterious	probably damaging	0	unknown	VCV000809640
c.1945A>T	p.Thr649Ser	M3	deleterious	probably damaging	0	Unknown	VCV002574317
c.1946C>T	p.Thr649Met	M3	deleterious	probably damaging	22	Unknown	VCV002053093
c.1948G>C	p.Ala650Pro	M3	deleterious	probably damaging	0	Unknown	VCV001316539
c.1949C>T	p.Ala650Val	M3	deleterious	probably damaging	0	Unknown	VCV001723717
c.1961C>G	p.Ala654Gly	M3	deleterious	probably damaging	0	Inborn genetic disease	VCV000985445
c.1981A>T	p.Ile661Phe	M3-S2	deleterious	probably damaging	96	Unknown	VCV000751483
c.2128C>T	p.Arg710Trp	S2	deleterious	probably damaging	13	Autosomal recessive spinocerebellar ataxia 18, inherited	VCV000427806
c.2138A>G	p.Asn713Ser	S2	tolerated	benign	16	Inborn genetic disease	VCV002230609
c.2141G>A	p.Arg714Gln	S2	tolerated	probably damaging	12	Inborn genetic disease	VCV002275090
c.2218G>A	p.Val740Ile	S2	deleterious	benign	327	Unknown	VCV000785104
c.2226T>G	p.Asp742Glu	S2	tolerated	probably damaging	6	Inborn genetic disease	VCV002369200
c.2302C>T	p.Arg768Trp	S2	deleterious	probably damaging	0	Unknown	VCV002162742
c.2328T>G	p.His776Gln	S2	tolerated	probably damaging	2	Unknown	VCV001950232
c.2489G>C	p.Ser830Thr	S2-M4	deleterious	benign	12	Unknown	VCV001991000
c.2585G>A	p.Arg862Gln	CTD	tolerated	probably damaging	15	Inborn genetic disease	VCV002212100
c.2609A>G	p.Lys870Arg	CTD	deleterious low confidence	probably damaging	0	Inborn genetic disease	VCV002352798
c.2824C>T	p.Arg942Cys	CTD	deleterious low confidence	probably damaging	3	Inborn genetic disease	VCV002380744
c.2825G>A	p.Arg942His	CTD	deleterious low confidence	probably damaging	7	Unknown	VCV002190998
c.2896A>G	p.Arg966Gly	CTD	deleterious low confidence	probably damaging	14	Unknown	VCV002058283
c.2921T>A	p.Phe974Tyr	CTD	tolerated	probably damaging	304	Unknown	VCV002050505
c.2975C>T	p.Thr992Ile	CTD	deleterious low confidence	benign	12	Unknown	VCV002180680
c.2995A>G	p.Asn999Asp	CTD	tolerated	benign	2	Inborn genetic disease	VCV002296698

Missense variants are named on the transcript NM_001510.4; only inherited or germline variants included. Missense variants that are absent from gnomAD are red. ClinVar was accessed on 9-15-23.

Supplemental Table S6. Predicted percent occupancy of GluD1-Cbln2 salt bridge interactions

GluD1			Cbln2			Percent occupancy
Residue	Residue #	Chain	Residue	Residue #	Chain	
Glu	58	H	Arg	181	F	100
Asp	21	H	Lys	212	F	100
Glu	58	H	Arg	204	D	99.5
Arg	341	H	Asp	176	D	99.5
Arg	341	G	Asp	178	A	99.5
Asp	21	G	Lys	212	B	97.2
Lys	357	H	Glu	107	F	95.8
Lys	357	G	Glu	104	B	60.5
Glu	58	G	Arg	204	A	47.4
Lys	59	G	Glu	203	A	24.7
Lys	357	G	Glu	107	B	20.5
Arg	353	H	Glu	104	F	0.93
Arg	341	H	Asp	178	D	0
Asp	21	H	His	103	F	0
His	344	G	Glu	104	B	0

All predicted salt bridge interactions between GluD1 and Cbln2 during MD simulation. Percent occupancy calculated using "Salt Bridges" VMD plugin. Identical residue pairs from different chains are calculated separately. Charged residues within 4 Å distance are considered occupied salt bridges.

Supplemental Table S7. Predicted percent occupancy of GluD1-Cbln2 hydrogen bond interactions

GluD1					Cbln2				
Residue	Residue #	Side/ Main	Donor/ Acceptor	Chain	Residue	Residue #	Side/ Main	Donor/ Acceptor	Percent occupancy
**Arg**	**341**	**S**	**D**	**G**	**Asp**	**178**	**S**	**A**	**97.21**
Glu	58	S	A	H	Arg	181	S	D	95.81
Glu	58	S	A	H	Arg	204	S	D	95.35
Arg	341	S	D	H	Asp	176	S	A	94.42
Glu	58	S	A	G	Arg	204	S	D	87.91
Glu	58	S	A	H	Tyr	153	S	D	77.68
Asp	21	S	A	H	Lys	212	S	D	77.21
Asp	21	S	A	H	Thr	101	S	D	69.77
Ser	348	S	D	H	Glu	104	S	A	65.58
Ser	345	M	D	H	Met	208	M	A	63.72
Lys	357	S	D	H	Glu	107	S	A	58.14
Asp	21	S	A	G	Lys	212	S	D	52.56
Ser	348	M	D	H	Glu	104	S	A	51.16
Arg	341	S	D	H	Asp	178	M	A	46.51
Ser	345	M	D	G	Met	208	M	A	45.12
Ser	345	S	D	H	Asn	102	M	A	43.26
Asp	21	S	A	G	Asn	102	S	D	41.4
His	344	S	D	G	Glu	104	S	A	41.4
Lys	357	S	D	G	Glu	104	S	A	35.95
Asn	360	S	D	H	Glu	107	S	A	34.42
Trp	343	M	A	G	Asn	154	S	D	30.7
Ser	345	S	D	G	Asn	102	M	A	30.23
Asp	21	S	A	H	Asn	102	M	D	26.05
Met	346	M	A	H	Glu	104	M	D	24.19
Asp	340	M	A	H	Arg	155	S	D	23.26
Trp	343	M	D	H	Tyr	153	M	A	22.79
Asp	21	S	A	G	Tyr	213	S	D	20
Met	346	M	A	G	Glu	104	M	D	19.53
Ala	20	M	D	G	Gln	159	S	A	17.67

Trp	343	M	D	G	Asn	154	S	A	14.88
Asn	360	S	D	G	Glu	104	S	A	14.88
Lys	59	S	D	G	Glu	203	S	A	14.88
Lys	357	S	D	G	Glu	107	S	A	13.02
Ser	345	S	D	G	Met	208	M	A	12.56
Arg	341	S	D	G	Asp	178	M	A	10.7
Ser	348	S	A	H	His	103	S	D	9.3
Ala	20	M	D	G	Glu	203	S	A	7.44
Gln	85	M	A	H	His	103	S	D	4.65
Asp	21	M	A	G	Tyr	153	S	D	3.26
Ala	20	M	D	G	Ser	171	S	A	2.79
Ala	20	M	A	G	Asn	102	S	D	2.79
Ser	348	S	A	G	His	103	S	D	2.33
Arg	341	M	A	G	Arg	155	M	D	2.33
Asp	53	S	A	G	Gln	177	S	D	1.86
Met	346	M	A	G	His	103	S	D	0.93
Ala	20	M	D	H	Ser	100	M	A	0.93
Arg	353	S	D	H	Glu	104	S	A	0.93
Asp	53	M	A	G	Gln	177	S	D	0.47
Asp	52	S	A	G	Gln	177	S	D	0.47
Asp	53	S	A	H	Gln	156	S	D	0.47
Asp	53	S	A	H	Gln	177	S	D	0.47

All predicted hydrogen bonding interactions between GluD1 and Cbln2 during MD simulation. Percent occupancy was calculated using the VMD "HBonds" plugin. Identical residue pairs from different chains are calculated separately. Donor-Acceptor distance was set to 3 Å and the hydrogen bonding angle cutoff was set to 20°. Whether the hydrogen bond interaction is occurring in a side/main chain is indicated with “S” or “M” for each of GluD1 and Cbln2. Hydrogen bond donors/acceptors are indicated with “D” or “A”.

Supplemental Table S8. Inhibitors of constitutively active GluD2 current

	**T649A IC_{50} [95% CI] (n)**	**A654T IC_{50} [95% CI] (n)**	**Fold-shift**
**D-Serine (1 mM Ca^{2+})**	0.69 mM [0.68,0.70] (5)	1.5 mM [1.4, 1.6] (7)	2.2
**Glycine**	0.24 mM [0.18,0.29] (11)	1.0 mM [0.94,1.2] (8)	4.4
**D-Alanine**	1.3 mM [0.44, 1.7] (9)	1.6 mM [1.3, 1.8] (12)	1.2
**L-Aspartic Acid**	0.27 mM [0.065, 0.3] (10)	5.0 mM [0.34, 17] (5)	19

Data are mean [95% CI Lower, Upper], with number of oocytes in parentheses (n). Fold-shift is IC_{50} A654T/IC_{50} T649A.

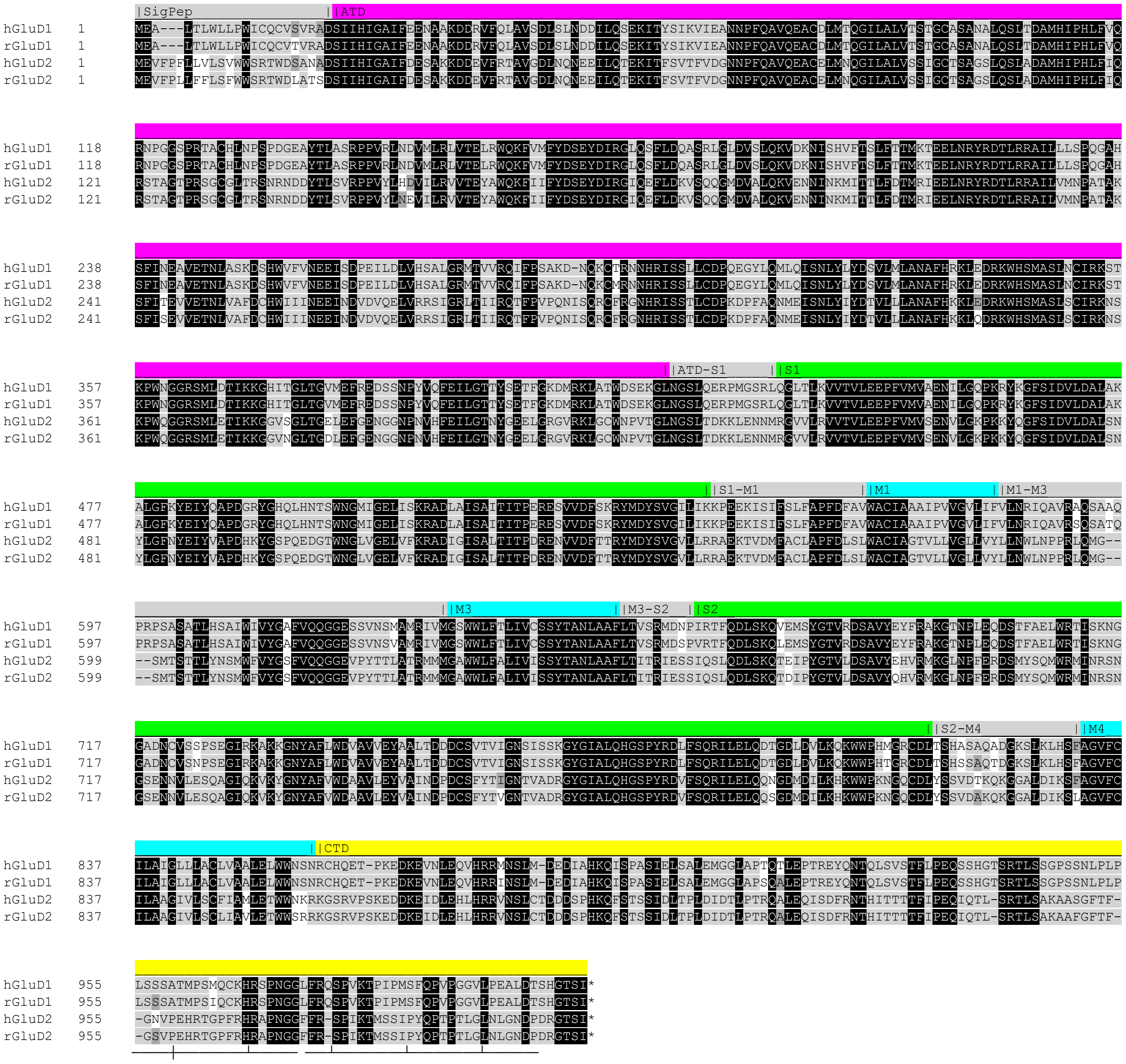

Supplemental Figure S1. Alignment of human and rat GluD1 and GluD2 protein sequences. Alignments of human GluD1 (NP_060021.1), rat GluD1 (NP_077354.1), human GluD2 (NP_001501.2), and rat GluD2 (NP_077355.1). Black: identical residues in all. Dark background indicates identical residues in all GluD1 and GluD2 (rat and human). Light gray background indicates identical residues in human GluD1 and GluD2, or in rat GluD1 and GluD2. Alignments made using Clustal Omega (EMBL-EBI). Domains based on RCSB PDB ID 6KSS (Burada et al. 2020).

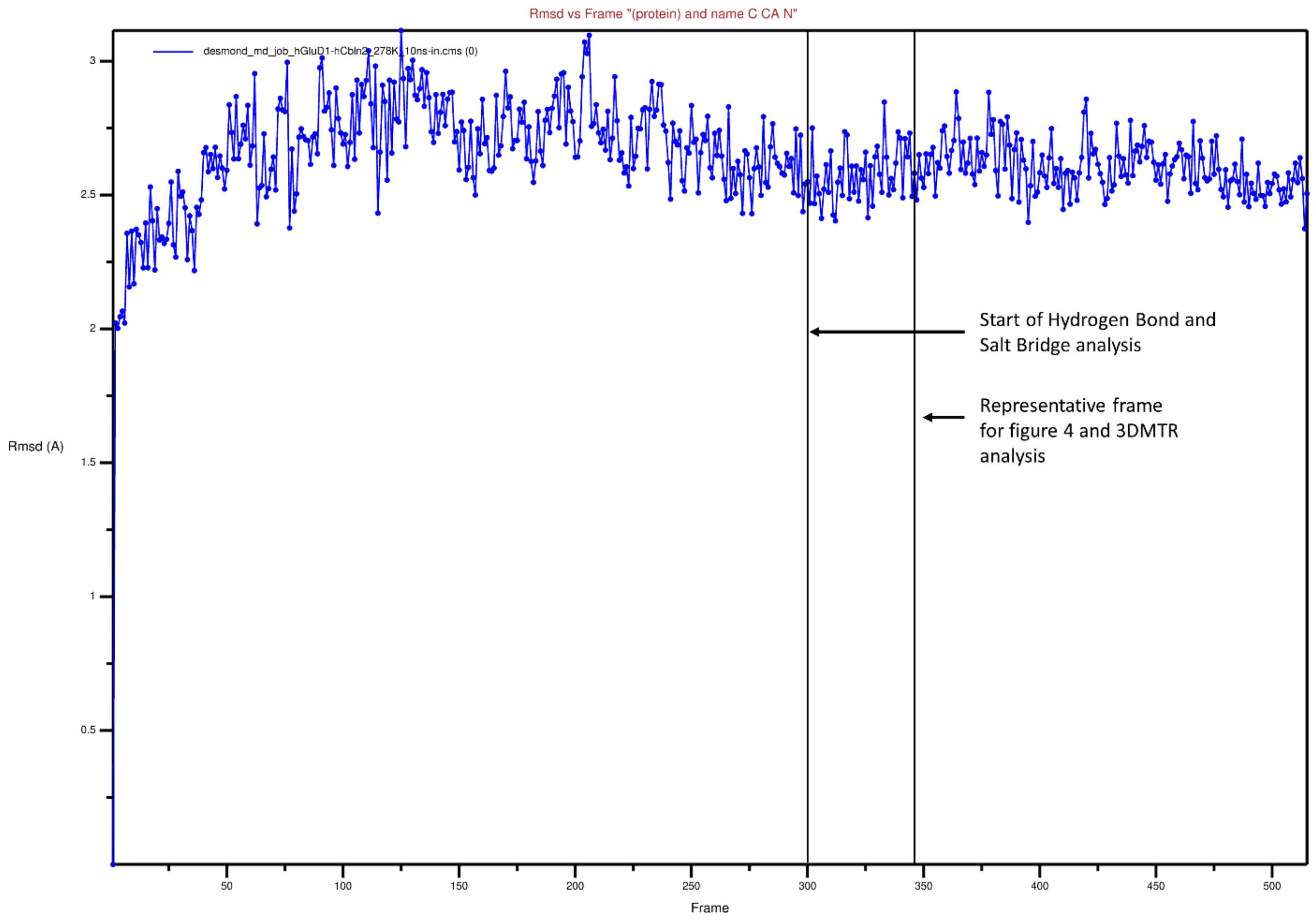

Supplemental Figure S2. RMSD of GluD1-Cbln2 homology model across MD simulation frames. Plot was generated using the VMD RMSD Trajectory tool. Salt bridge and hydrogen bond percent occupancy analysis was performed after RMSD stabilization (Table S6, S7). The representative frame used to generate Figure 4 of the main text is indicated.

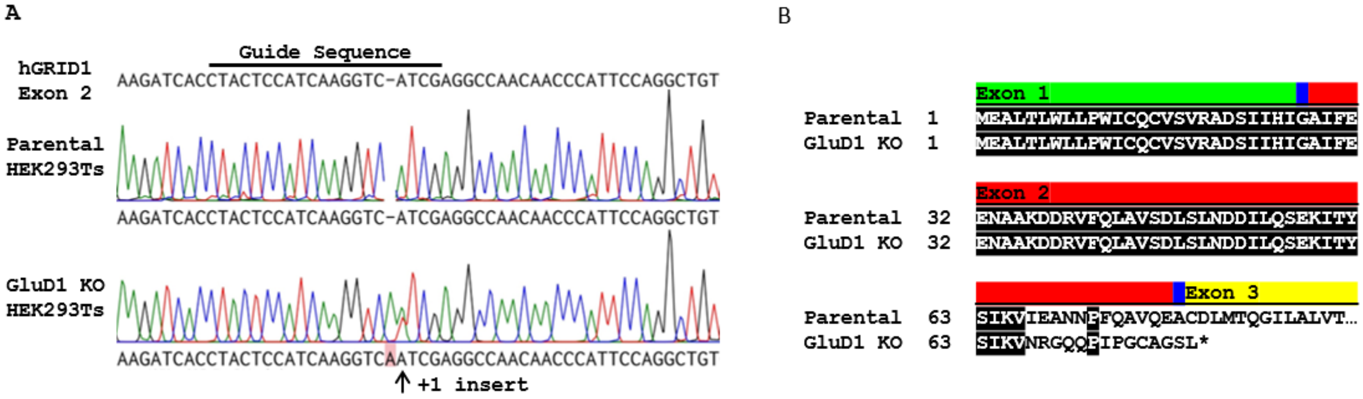

Supplemental Figure S3. Genotype of the GluD1 KO cell line. (A) Sequence alignment and Sanger sequencing traces of the parental HEK293T and GluD1 KO cell lines demonstrating a +1 A/T insertion into the second exon of the *GRID1* gene immediately following the red highlighted base. **(B)** The predicted *GRID1* gene product in the parental (wild type) and GluD1 KO cell lines. The +1 insertion induces frame shift at amino acid 67 of the GluD1 polypeptide chain, producing a mutant C-terminal sequence before prematurely terminating within exon 3. A black background indicates identical residues in both proteins.

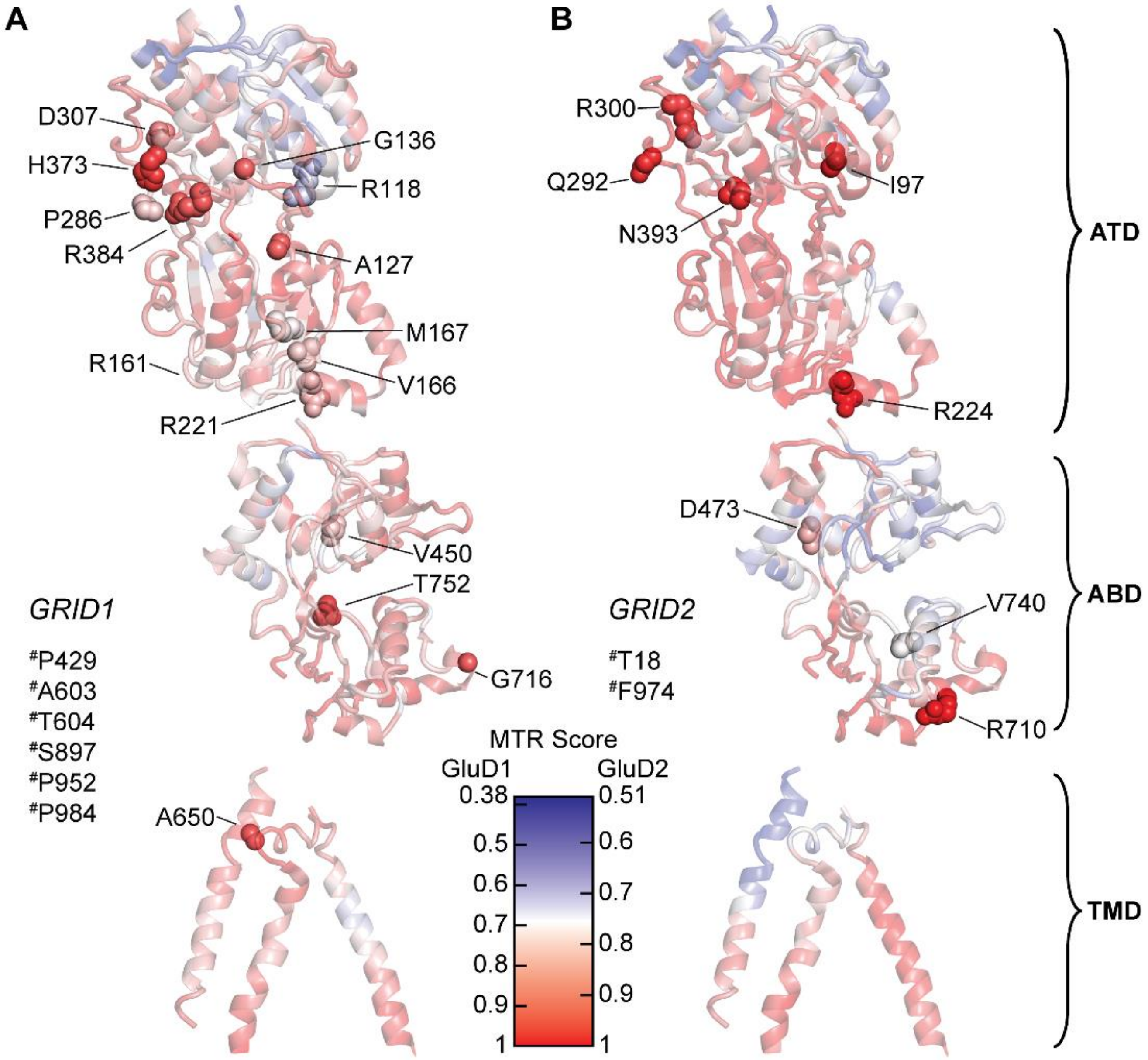

Supplemental Figure S4. 3D MTR analysis of other *GRID1* and *GRID2* variants. 3D MTR for missense variants in Supplemental Tables S2-S5 that were present in the gnomAD database are primarily localized in tolerant domains. 3D MTR scores were obtained using the GluD1 protein structure (Burada et al., 2020b) (PDB ID 6KSS). Lower scores (Blue) represent less tolerant residues, while higher scores (Red) represent more tolerant residues. **(A)** *GRID1* variants in context of *GRID1* 3D MTR scores. **(B)** *GRID2* variants in context of *GRID2* 3D MTR scores. # indicates missense variants in gnomAD that reside in regions of the receptor for which there is not clear electron density in the structure.

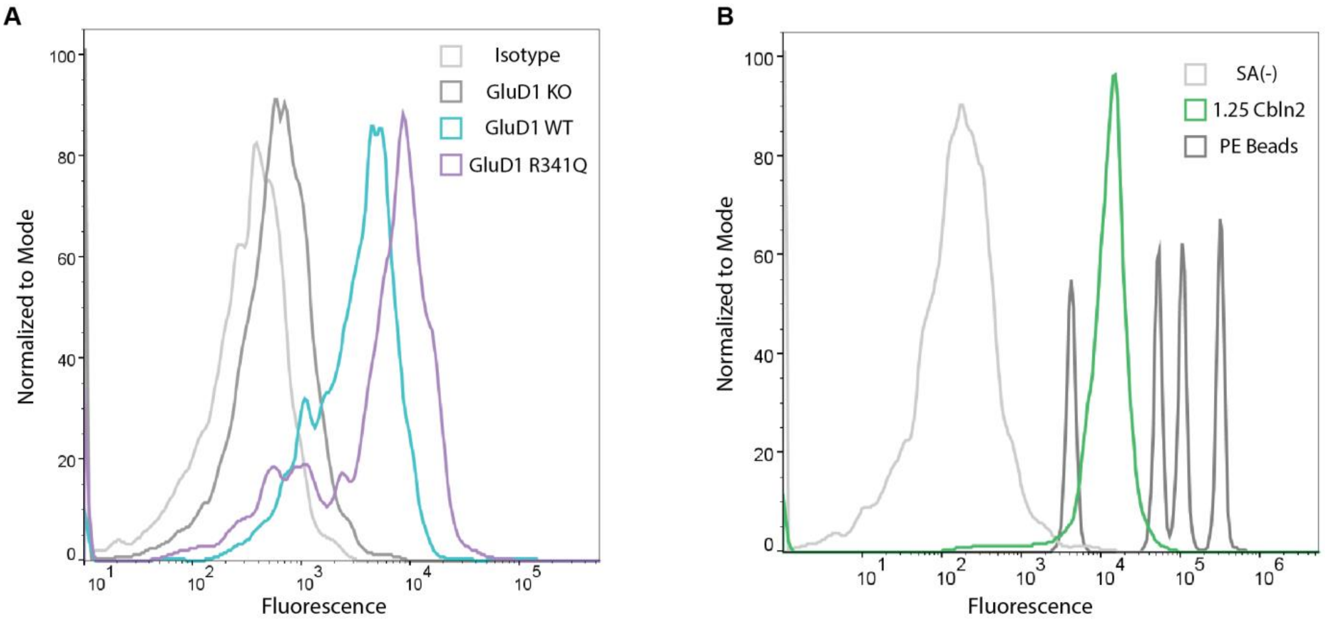

Supplemental Figure S5. Flow cytometry validation of GluD1 and Cbln2 presence.
(A) Normalized fluorescence histogram showing similar expression levels of the GluD1 between the WT and R341Q in transduced HEK cells, stained with primary rabbit anti-GluD1 antibody and secondary anti-rabbit AF647 antibody. GluD1 KO cells were stained the same way and the isotype control was for the rabbit anti-GluD1 antibody. (**B)** Beads coated with either 1.25 μg/mL Cbln2 or 0 μg/mL Cbln2 (SA(-) Ctrl) were stained with anti-HIS PE antibody to validate presence of Cbln2 on the bead used for making probes in the BFP adhesion frequency curve. BD science quantum PE calibration beads (PE beads) were flowed simultaneously for reference values.

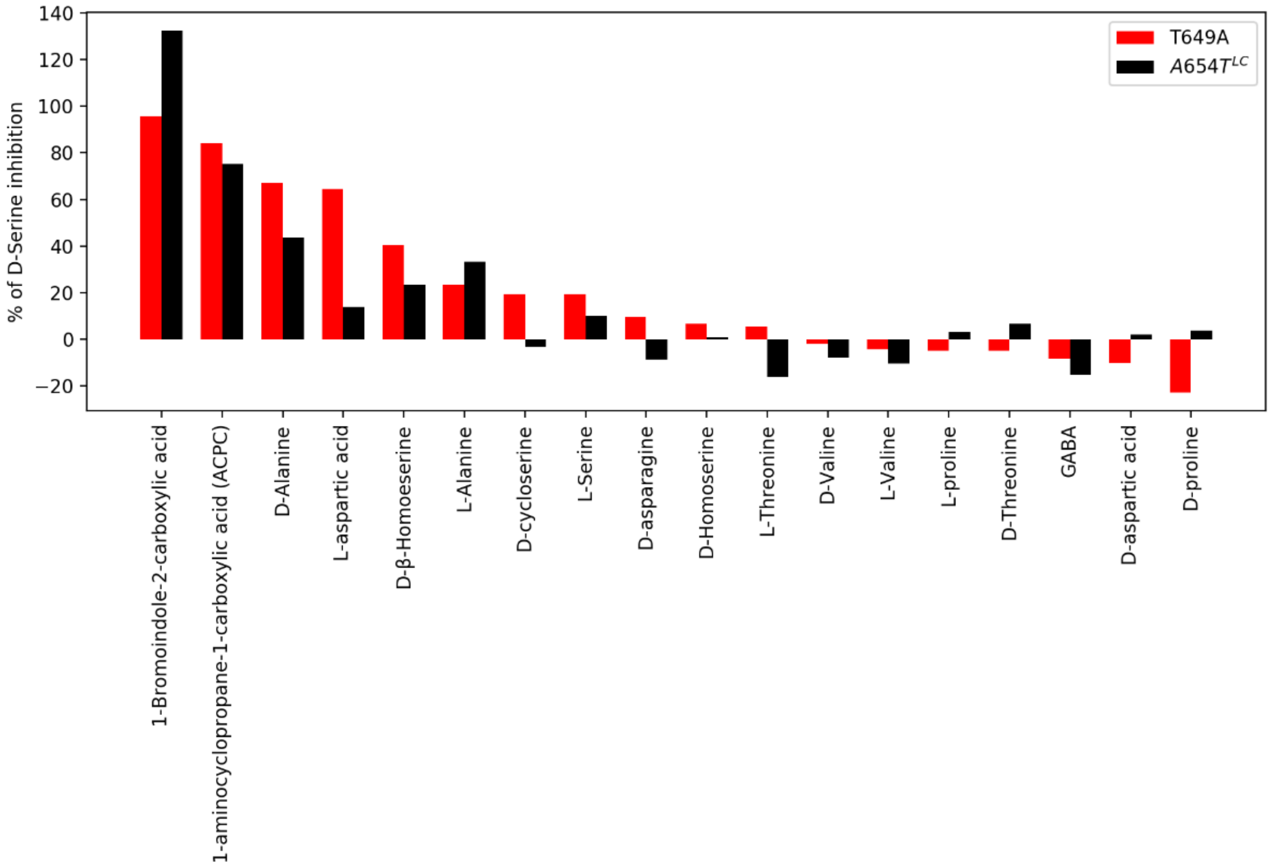

Supplemental Figure S6. Single-concentration screen of compounds on GluD1-T649A and GluD2-A654T in oocytes. All compound were tested at a concentration of 1 mM. Results are displayed as a percent of inhibition obtained with 1 mM D-serine in the same recording. 2 oocytes were tested for each compound and variant. GluD2-A654T^{LC} indicates the missense variant in the *lurcher* mouse model.

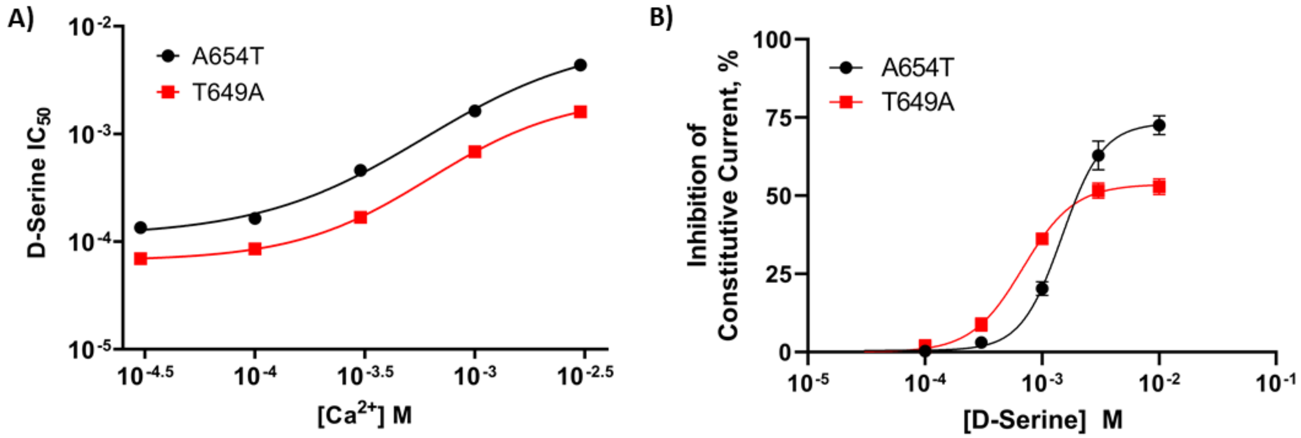

Supplemental Figure S7. Allosteric interactions of D-serine and Ca^{2+} in GluD2-T649A. (A) [Ca^{2+}] concentration *vs* D-serine IC_{50} is shown and fitted by the Hill equation. There is a relationship between Ca^{2+} concentration and the D-Serine IC_{50}, with D-serine being more potent at GluD2-T649A than at the *lurcher* mutation (GluD2-A654T) for all concentrations of extracellular Ca^{2+}. Fitted parameters for GluD2-A654T were Ca^{2+} EC_{50} 590 µM, Hill slope 1.1, Minimum 110 µM, Maximum 7500 µM. Fitted parameters for GluD2-T649A were EC_{50} 630 µM, Hill slope 1.4, Minimum 66 µM, Maximum 2300 µM. **(B)** D-Serine concentration-response curve reveals that D-serine shows increased potency for GluD2-T649A in 1 mM Ca^{2+}. Fitted parameters for GluD2-A654T were D-serine EC_{50} 1500 µM, Hill slope 2.5, Maximum 73%. Fitted parameters for GluD2-T649A were D-serine EC_{50} 690 µM, Hill slope 2.0, Maximum 54%.

References

Ali, Z., Zulfiqar, S., Klar, J., Wikstrom, J., Ullah, F., Khan, A., Abdullah, U., Baig, S., Dahl, N. (2017). Homozygous GRID2 missense mutation predicts a shift in the D-serine binding domain of GluD2 in a case with generalized brain atrophy and unusual clinical features. *BMC Med Genet, 18*(1), 144.

Burada, A. P., Vinnakota, R., & Kumar, J. (2020). Cryo-EM structures of the ionotropic glutamate receptor GluD1 reveal a non-swapped architecture. *Nat Struct Mol Biol*, 27(1), 84-91.

Coutelier, M., Burglen, L., Mundwiller, E., Abada-Bendib, M., Rodriguez, D., Chantot-Bastaraud, S., Rougeot, C., Cournelle, M.A., Milh, M., Toutain, A., Bacq, D., Meyer, V., Afenjar, A., Deleuze, J.F., Brice, A., Héron, Stevanin, G., Durr, A. (2015). *GRID2* mutations span from congenital to mild adult-onset cerebellar ataxia. *Neurology, 84*(17), 1751-1759.

Fogel, B. L., Lee, H., Deignan, J. L., Strom, S. P., Kantarci, S., Wang, X., Quintero-Rivera, F., Vilain, E., Grody, W. W., Perlman, S., Geschwind, D. H., & Nelson, S. F. (2014). Exome sequencing in the clinical diagnosis of sporadic or familial cerebellar ataxia. *JAMA neurology, 71*(10), 1237–1246.

Hills, L. B., Masri, A., Konno, K., Kakegawa, W., Lam, A. T., Lim-Melia, E., Chandy, N., Hill, R.S., Partlow, J.N., Al-Saffar, M., Nasir, R., Stoler, J.M., Barkovich, A.J., Watanabe, M., Yuzaki, M., Mochida, G. H. (2013). Deletions in *GRID2* lead to a recessive syndrome of cerebellar ataxia and tonic upgaze in humans. *Neurology* 81(16), 1378-1386.

Kushima, I., Aleksic, B., Nakatochi, M., Shimamura, T., Okada, T., Uno, Y., Morikawa, M., Ishizuka, K., Shiino, T., Kimura, H., Arioka, Y., Yoshimi, A., Takasaki, Y., Yu, Y., Nakamura, Y., Yamamoto, M., Iidaka, T., Iritani, S., Inada, T., Ogawa, N., … Ozaki, N. (2018). Comparative Analyses of Copy-Number Variation in Autism Spectrum Disorder and Schizophrenia Reveal Etiological Overlap and Biological Insights. *Cell Reports*, 24(11), 2838-2856.

Maier, A., Klopocki, E., Horn, D., Tzschach, A., Holm, T., Meyer, R., & Meyer, T. (2014). De novo partial deletion in GRID2 presenting with complicated spastic paraplegia. *Muscle & nerve*, 49(2), 289–292.

Petrovski, S., Wang, Q., Heinzen, E.L., Allen, A.S., Goldstein, D.B. (2013) Genic intolerance to functional variation and the interpretation of personal genomes. *PLoS Genet*. 9(8):e1003709.

Taghdiri, M., Kashef, A., Abbassi, G., Moshtagh, A., Sadatian, N., Fardaei, M., Najafi, K., & Kariminejad, R. (2019). Further delineation of the phenotype caused by a novel large homozygous deletion of *GRID2* gene in an adult patient. *Clinical case reports, 7*(6), 1149-1153.

Ung, D.C., Tricoire, L. Pietrancosta, N., Zlatanovic, A., Pode-Shakked, B., Raas-Rothschild, A., Elpeleg, O., Abu-Libdeh, B., Hamed, N., Papon, M-A, Marouillat, S., Thepault, R-A, Stevanin, G., Lambolez, B., Toutain, A., Hepp, R., Laumonnier, F. (2022) GRID1/GluD1 homozygous variants linked to intellectual disability and spastic paraplegia impair mGlu1/5 receptor signaling and excitatory synapses. medRxiv, https://doi.org/10.1101/2022.05.16.22274994

Utine, G. E., Haliloğlu, G., Salanci, B., Çetinkaya, A., Kiper, P. Ö., Alanay, Y., Aktas, D., Boduroğlu, K., & Alikaşifoğlu, M. (2013). A homozygous deletion in *GRID2* causes a human phenotype with cerebellar ataxia and atrophy. *J Child Neurol*, 28(7), 926-932.

Van Schil, K., Meire, F., Karlstetter, M., Bauwens, M., Verdin, H., Coppieters, F., Scheiffert, E., Van Nechel, C., Langmann, T., Deconinck, N., & De Baere, E. (2015). Early-onset autosomal recessive cerebellar ataxia associated with retinal dystrophy: new human hotfoot phenotype caused by homozygous *GRID2* deletion. *Genetics in Medicine*, 17(4), 291-299.

Veerapandiyan, A., Enner, S., Thulasi, V., & Ming, X. (2017) A Rare Syndrome of *GRID2* Deletion in 2 Siblings. *Child Neurol Open*, 4: 1-3
